# The RBM14/CoAA-interacting, long intergenic non-coding RNA *Paral1* regulates adipogenesis and coactivates the nuclear receptor PPARγ

**DOI:** 10.1038/s41598-017-14570-y

**Published:** 2017-10-26

**Authors:** François F. Firmin, Frederik Oger, Céline Gheeraert, Julie Dubois-Chevalier, Anne-Sophie Vercoutter-Edouart, Fawaz Alzaid, Claire Mazuy, Hélène Dehondt, Jeremy Alexandre, Bruno Derudas, Quentin Dhalluin, Maheul Ploton, Alexandre Berthier, Eloise Woitrain, Tony Lefebvre, Nicolas Venteclef, François Pattou, Bart Staels, Jérôme Eeckhoute, Philippe Lefebvre

**Affiliations:** 1 Univ. Lille, Inserm, CHU Lille, Institut Pasteur de Lille, U1011- EGID, F-59000 Lille, France; 2 CNRS, UMR 8576, UGSF, Unité de Glycobiologie Structurale et Fonctionnelle, FRABio FR 3688, Univ, Lille, Villeneuve d’Ascq, F-59650 France; 3 INSERM UMRS 1138, Sorbonne Universités, UPMC Université Paris 06; Sorbonne Paris Cité, Université Paris Descartes, Université Paris Diderot; and Centre de Recherche des Cordeliers, Paris, F-75006 France; 4 Univ. Lille, Inserm, CHU Lille, U1190- EGID, F-59000 Lille, France

## Abstract

Adipocyte differentiation and function relies on a network of transcription factors, which is disrupted in obesity-associated low grade, chronic inflammation leading to adipose tissue dysfunction. In this context, there is a need for a thorough understanding of the transcriptional regulatory network involved in adipose tissue pathophysiology. Recent advances in the functional annotation of the genome has highlighted the role of non-coding RNAs in cellular differentiation processes in coordination with transcription factors. Using an unbiased genome-wide approach, we identified and characterized a novel long intergenic non-coding RNA (lincRNA) strongly induced during adipocyte differentiation. This lincRNA favors adipocyte differentiation and coactivates the master adipogenic regulator peroxisome proliferator-activated receptor gamma (PPARγ) through interaction with the paraspeckle component and hnRNP-like RNA binding protein 14 (RBM14/NCoAA), and was therefore called PPARγ-activator RBM14-associated lncRNA (*Paral1*). *Paral1* expression is restricted to adipocytes and decreased in humans with increasing body mass index. A decreased expression was also observed in diet-induced or genetic mouse models of obesity and this down-regulation was mimicked *in vitro* by TNF treatment. In conclusion, we have identified a novel component of the adipogenic transcriptional regulatory network defining the lincRNA *Paral1* as an obesity-sensitive regulator of adipocyte differentiation and function.

## Introduction

White adipose tissue (WAT) is a dynamic organ responding to dietary intakes by a rapid morphological remodeling whose kinetics depends on WAT localization within the body^1^. Expanding WAT mass stores energy in periods of plenty and is a safeguard against lipid accumulation in peripheral tissues, a major contributor to insulin resistance and associated co-morbidities such as type 2 diabetes (T2D)^2^. Indeed, increased fat deposition in WAT may be protective and metabolic health thus relies in part on WAT expandability, which depends on WAT hyperplasia and adipocyte hypertrophy^3^. In the context of obesity, hypertrophied adipocytes are prone to cell death^4^, hence triggering macrophage infiltration and TNF-induced PPARγ downregulation among other processes^5^. Furthermore, adipocyte size positively correlates with insulin resistance and T2D and is thus pathologically meaningful^6^. In contrast, WAT hyperplasia is metabolically more beneficial than hypertrophy^7^.

De novo adipogenesis, leading to WAT hyperplasia, is thus required for WAT to cope with a positive energy balance. Adipogenesis is a highly complex mechanism relying on the sequential activation or repression of transcriptional regulators leading to a mature lipid-storing adipocyte phenotype. The core of the terminal differentiation signaling pathway is constituted by the transcription factor CCAATT enhancer-binding protein β (C/EBPβ) which regulates the expression of PPARγ^8^ and of C/EBPα^9^. The coordinated interplay of these 2 transcription factors triggers complex epigenomic remodeling to achieve adipocyte maturation^8,10–12^.

Pervasive transcriptional events throughout the genome generate numerous RNA transcripts without protein coding potential [non-coding (nc) RNAs] and covering ~60% of the genome. Among those, long non-coding RNAs (lncRNAs, > 200 nt) play a role in diverse biological processes such as cellular differentiation^13,14^. LncRNAs are expressed in a highly tissue-specific manner and display a wide array of functions in the cytoplasm and/or the nucleus often related to transcriptional and post-transcriptional gene regulation, as well as to organization of chromosome and nucleus topology^15,16^. Considering their generally low abundance and cell-specific expression, lncRNAs have also been proposed to be mere by-products of transcription which is a nuclear structure-regulatory event per se^17^.

Several lncRNAs (*Neat1*, *Adinr* and *lnc-U90926*) interfere with terminal adipocyte differentiation by modulating PPARγ or C/EBPα expression^18–20^. The exact molecular mechanisms involved in lncRNA-mediated control of adipogenesis remain however poorly defined. As no investigation of their possible contribution to WAT physiopathology has been reported, a case-by-case investigation remains necessary to decipher the mechanism of action of lncRNAs.

In this study, we characterized a novel adipocyte-specific lincRNA potentiating the adipogenic function of PPARγ through interaction with RNA Binding Motif Protein 14 (RBM14/CoAA), hereafter called *Paral1* for PPARγ-activator RBM14-associated lncRNA. Loss-of-function experiments demonstrated its positive contribution to adipocyte differentiation. Expression studies in obese mice and humans showed a similarly decreased expression of *Paral1* in obese WAT, thereby identifying a novel adipogenic pathway dysregulated in obesity.

## Results

### *Paral1* is a long intergenic non-coding RNA specifically expressed in mature white adipocytes

**Figure 1:**
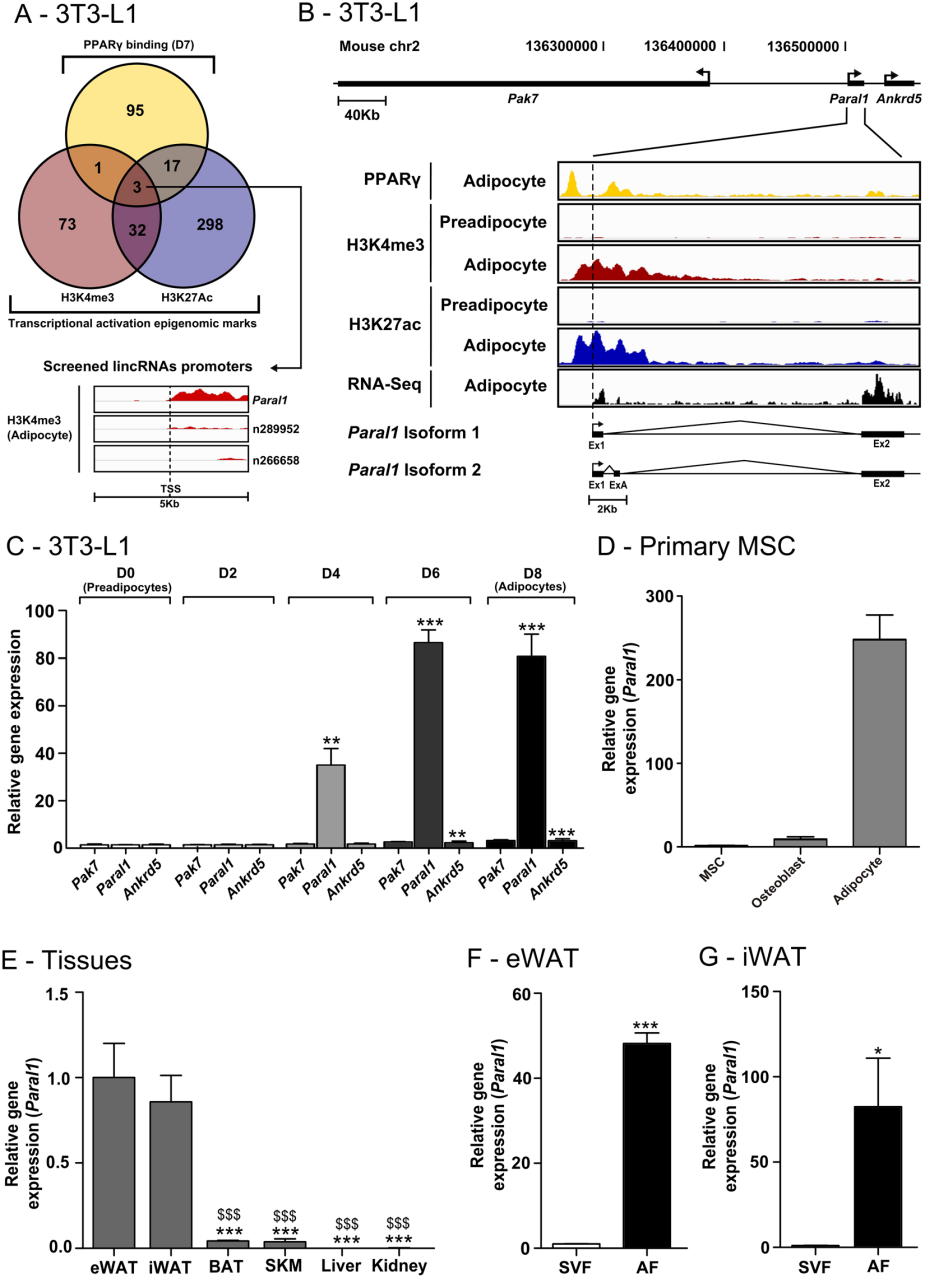
Identification of *Paral1*, an adipocyte-specific lincRNA. (**A**) Identifying long intergenic non-coding (linc) RNA promoter regions activated during 3T3-L1 adipogenesis. LincRNA promoter regions were scanned for increasing ChIP-seq signals for H3K4me3 (red) or H3K27Ac (blue) upon 3T3-L1 cell differentiation as well as PPARγ binding in adipocytes (yellow). The resulting Venn diagram shows signal overlaps between the identified lincRNA promoters. Numbers indicate the number of transcripts potentially regulated by identified promoters. Lower inset: H3K4me3 profiles in 3T3-L1 adipocytes for the 3 identified lincRNAs (Noncode v.3). (**B**) ChIP-seq profiles at the *Paral1* locus. PPARγ-, H3K4me3- and H3K27-enriched sequences were visualized using the IGV browser, as well as the RNA-seq profile allowing the identification of *Paral1* exons (lower panel). (**C**) *Paral1* transcript level in differentiating 3T3-L1 cells. *Paral1* expression was monitored at indicated times by RT-qPCR and normalized against the reference *Rplp0* housekeeping gene expression level. The expression of neighboring genes (5’: *Pak7*, 3′: *Ankrd5*) was assayed similarly. Results are expressed as the mean ± S.E.M. (n = 3) relative to *Paral1* RNA level in preadipocytes arbitrarily set to 1. The statistical significance of differences was established using a 1-way ANOVA followed by a Dunnett post hoc test. *p < 0.05; **p < 0.01; ***p < 0.001. (**D**) *Paral1* expression in mouse mesenchymal stem cells (MSCs). MSCs were differentiated for 14 days and *Paral1* expression was assessed by RT-qPCR as in (**C**). (**E**) *Paral1* expression in mouse tissues. *Paral1* transcript levels in indicated mouse tissues were assayed by RT-qPCR as above. The statistical significance of differences was established using a 1-way ANOVA followed by a Tukey post hoc test. Comparing to eWAT control: *p < 0.05; **p < 0.01; ***p < 0.001. Comparing to iWAT control: ^$^p < 0.05; ^$$^p < 0.01; ^$$$^p < 0.001. eWAT: epididymal white adipose tissue, iWAT: inguinal white adipose tissue, BAT: brown adipose tissue, SKM: skeletal muscle. (**F**,**G**) *Paral1* expression level in fractionated adipose tissues. *Paral1* expression was measured in the stromal vascular fraction (SVF) and adipocyte fraction (AF) from epididymal (eWAT, **F**) and inguinal (iWAT, **G**) white adipose tissues (WAT).

To identify lincRNA(s) expressed in adipose tissue and regulated during adipogenesis, we mined the NONCODE v3.0 database (http://www.noncode.org) containing 36,991 lncRNAs, from which 9,364 lincRNAs could be identified by filtering out transcripts overlapping with RefSeq genes. Using NGS data from differentiating 3T3-L1 cells^21^, a well-established model for adipocyte differentiation, 406 lincRNAs from the NONCODE database displaying an increased density in H3K4me3 and H3K27ac ChIP-seq signals within +/− 2.5 kb from the TSS upon differentiation were identified (Supplemental Table 2, Fig. 1A). Additional filtering using PPARγ ChIP-Seq signals narrowed this list down to 3 lincRNAs, amongst which *BC034902*, hereafter termed *Paral1* (PPARγ-activator RBM14-associated lincRNA 1), displayed the strongest levels of transcriptional activation marks (Fig. 1A, lower inset, and Fig. 1B). This 2.4 kb transcript is devoid of strong coding potential (Supplemental Table 3) and may occur as 2 isoforms in 3T3-L1 cells, of which isoform 1 is predominantly expressed (Fig. 1B, Supplemental Fig. 1). The 2 flanking protein-coding genes *Pak7* and *Ankrd5* genes display no histone activating marks neither in 3T3-L1 cells (Supplemental Fig. 2A) nor in primary adipocytes (Supplemental Fig. 2B) and are poorly activated during 3T3-L1 differentiation (Fig. 1C). This suggests that *Paral1* is an autonomous transcription unit not stemming from spurious read-through processes. In contrast, *Paral1* expression was potently induced during 3T3-L1 [fold change (FC = 70)], Fig. 1C) and 3T3-F442A differentiation (FC = 25, Supplemental Fig. 3). Murine mesenchymal stem cell (MSC) differentiation toward the adipocyte lineage was equally accompanied by a strong upregulation of *Paral1* (FC = 250), in contrast to osteoblastic differentiation during which *Paral1* expression was not modified compared to osteoblastic markers (*Runx2*, *Osteocalcin*) [Fig. 1D, Supplemental Fig. 4]. *Paral1* expression was restricted to mouse white adipose tissue (WAT) (Fig. 1E). *Paral1* was almost exclusively detected in mature adipocytes (AF) but not in the stromal vascular fraction (SVF) (Fig. 1F,G), in line with the data on *in vitro* differentiated adipocytes (Fig. 1C) and with the specific marking of the *Paral1* promoter with H3K4me3 and H3K27ac in isolated adipocytes (Supplemental Fig. 2B). *Paral1* expression is therefore markedly restricted to adipocytes and increases during adipocyte differentiation.

### *Paral1* is required for adipocyte differentiation

**Figure 2:**
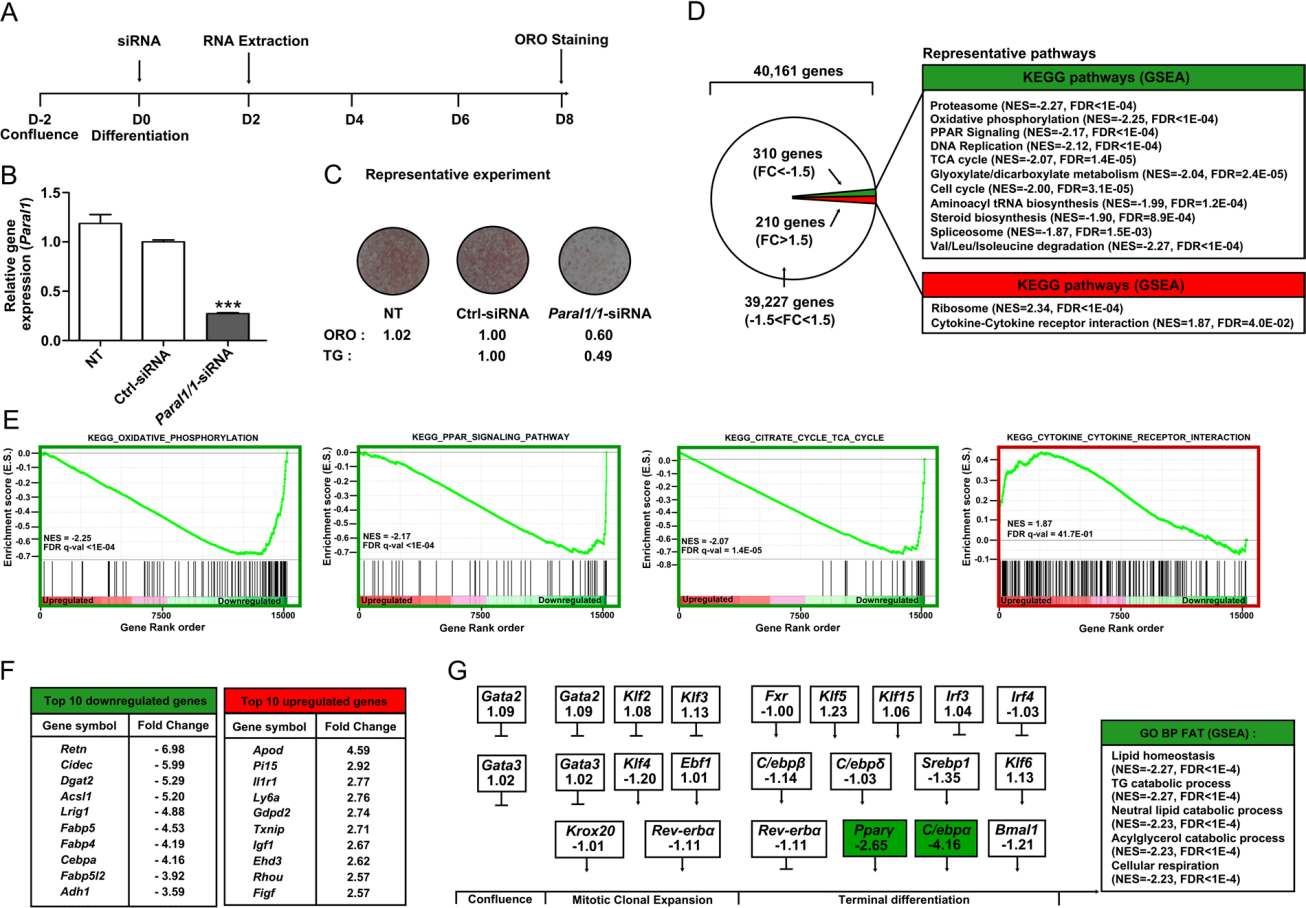
Paral1 expression is required for adipocyte differentiation. (**A**) 3T3-L1 differentiation and transfection protocol. B) Validation of *Paral1* expression knockdown. 3T3-L1 cells were transfected with control (Ctrl-siRNA) or *Paral1*-targeting siRNA (*Paral1/1*-siRNA) at D0 and differentiation was initiated at the same time. *Paral1* transcripts were assayed by RT-qPCR at D2 and results are expressed as the mean ± S.E.M. (n = 3) relative to the *Paral1* RNA level in Ctrl-siRNA transfected preadipocytes arbitrarily set to 1. Values were compared using a 1-way ANOVA followed by a Dunnett post hoc test. *p < 0.05; **p < 0.01; ***p < 0.001. NT: non transfected preadipocytes. (**C**) Lipid accumulation in differentiated adipocytes. Oil Red O staining of 3T3L1 cells was performed at D8. Numbers indicate intracellular ORO stain and intracellular triglycerides quantification relative to non-transfected (NT) cells for a representative experiment. (**D**) Knockdown of *Paral1* expression alters the expression of a specific subset of genes. The 3T3-L1 cell transcriptome was characterized by DNA microarray analysis as described in the Materials & Methods section. GSEA was performed against the KEGG database. NES: normalized enrichment score, FDR: false discovery rate), red: up-regulated after *Paral1/1*-siRNA treatment, green: down-regulated after *Paral1/1*-siRNA treatment. (**E**) GSEA enrichment plots of oxidative phosphorylation, TCA cycle, PPAR signaling pathway and cytokine-cytokine receptor signature genes in differentiating 3T3-L1 cells. Green curves depict the enrichment score for each gene (vertical black line) ranked along a heatmap (red to green, up- to down-regulated genes) indicating the observed fold change in *Paral1/1*-siRNA treated cells. NES: normalized enrichment score, FDR: false discovery rate. (**F**) Top-ranking genes in *Paral1/1*-siRNA treated cells. (**G**) Expression level variation of major contributors to the adipogenic program and top ranking biological theme (using the gene ontology biological process functional annotation table, GO BP FAT) of down-regulated genes in *Paral1*-depleted cells.

In vitro loss-of-function experiments (Fig. 2A) were carried out in 3T3-L1 cells by transfecting a siRNA targeting the *Paral1* transcript (Paral1/1-siRNA). A decreased expression (>70%, Fig. 2B) corresponded to a significantly blunted lipid storage (Fig. 2C) and decreased adipocyte-specific gene expression (Supplemental Fig. 5). Transfection with other siRNA targeting *Paral1* (Paral1/2-siRNA) similarly showed an impact on adipogenesis (Supplemental Fig. 6). The most efficient siRNA (Paral1/1-siRNA) was selected for further experiments. Silencing of the neighbouring gene *Ankrd5*, whose expression is modestly increased during differentiation (Fig. 1C), did not impact on lipid storage (Supplemental Fig. 7), indicating that *Paral1* does not act through regulation of this flanking gene. To identify regulatory pathway(s) controlled by *Paral1*, we compared gene expression patterns in differentiating adipocytes (D2) depleted or not of *Paral1*. This allowed the identification of ~500 genes deregulated upon *Paral1* silencing (Fig. 2D). Importantly, the expression of genes associated with white adipogenesis (*AdipoQ*, *aP2*) was decreased, in contrast to genes associated with brown adipogenesis (*Ebf2*, *Prdm16*, *Ucp1*) which remained unchanged (Supplemental Table 4). Gene set enrichment analysis (GSEA) revealed that many down-regulated genes are associated with oxidative metabolism and PPAR signalling, whereas up-regulated genes are functionally related to protein synthesis and cytokine signalling (Fig. 2E,F). Additional term enrichment analysis based on the Gene Ontology “Biological Process” functional annotation table (GO BP FAT) indicated that down-regulated genes are involved in lipid homeostasis and metabolism (Fig. 2G). This is in line with a role for *Paral1* in the acquisition of the mature adipocyte phenotype, as indicated by the altered lipid-storing capacity of *Paral1*-depleted 3T3-L1 cells (Fig. 2C). A significant and selective down-regulation of the adipogenic master genes *Pparγ* and *C/ebpα* was observed (Fig. 2G and Supplemental Table 5). *Paral1* knockdown using an unrelated LNA gapmer generated a similar phenotype, confirming the role of *Paral1* in adipogenesis (Supplemental Fig. 8). However, inducible *Paral1* overexpression did not promote 3T3-L1 cell differentiation on its own, in contrast to *Pparγ* overexpression (Supplemental Fig. 9). Taken as a whole, our data show that *Paral1* is necessary, but not sufficient in our conditions, for terminal adipocyte differentiation.

### *Paral1* contributes to adipocyte phenotype maintenance

We next assessed whether *Paral1* is required to maintain a fully mature adipocyte phenotype. Silencing of *Paral1* in differentiated 3T3-L1 adipocytes at D7 (Supplemental Fig. 10A,B) perturbed the expression of a minor fraction of transcripts (∼100 genes, FC > 1.5, p < 0.05). A gene set enrichment analysis of expression data emphasized the down-regulation of genes involved in functions similar to those previously identified upon *Paral1* silencing in differentiating cells (D2) and notably including oxidative phosphorylation (Supplemental Fig. 10C). A gene-by-gene analysis showed *Paral1* depletion affected neither *Pparγ* nor *C/ebpα* expression (Supplemental Fig. 10E) but reduced that of genes participating to glycerolipid and cholesterol synthesis (*Agpat2*, *Gpam*, *Lpin1*, *Cyp51a1*, *Lss*…). These biosynthetic pathways are not only under the tight control of PPARγ but are also coordinately regulated by SREBP1c, CHREBP and/or LXR. *Paral1* depletion affected mostly PPARγ target genes (Supplemental Fig. 10E). However, the expression of several other bona-fide direct PPARγ target genes such as *aP2/Fabp4*, *Cd36* or *Glut4* was left unchanged in these conditions. Whether this results from distinct regulatory mechanisms or from a differential sensitivity to *Paral1* depletion remains to be established. Taken together, these data however suggest that *Paral1* sustains at least in part PPARγ transcriptional activity in mature adipocytes.

### *Paral1* localization is mainly nuclear and interacts with the transcriptional coactivator and paraspeckle component RBM14

**Figure 3:**
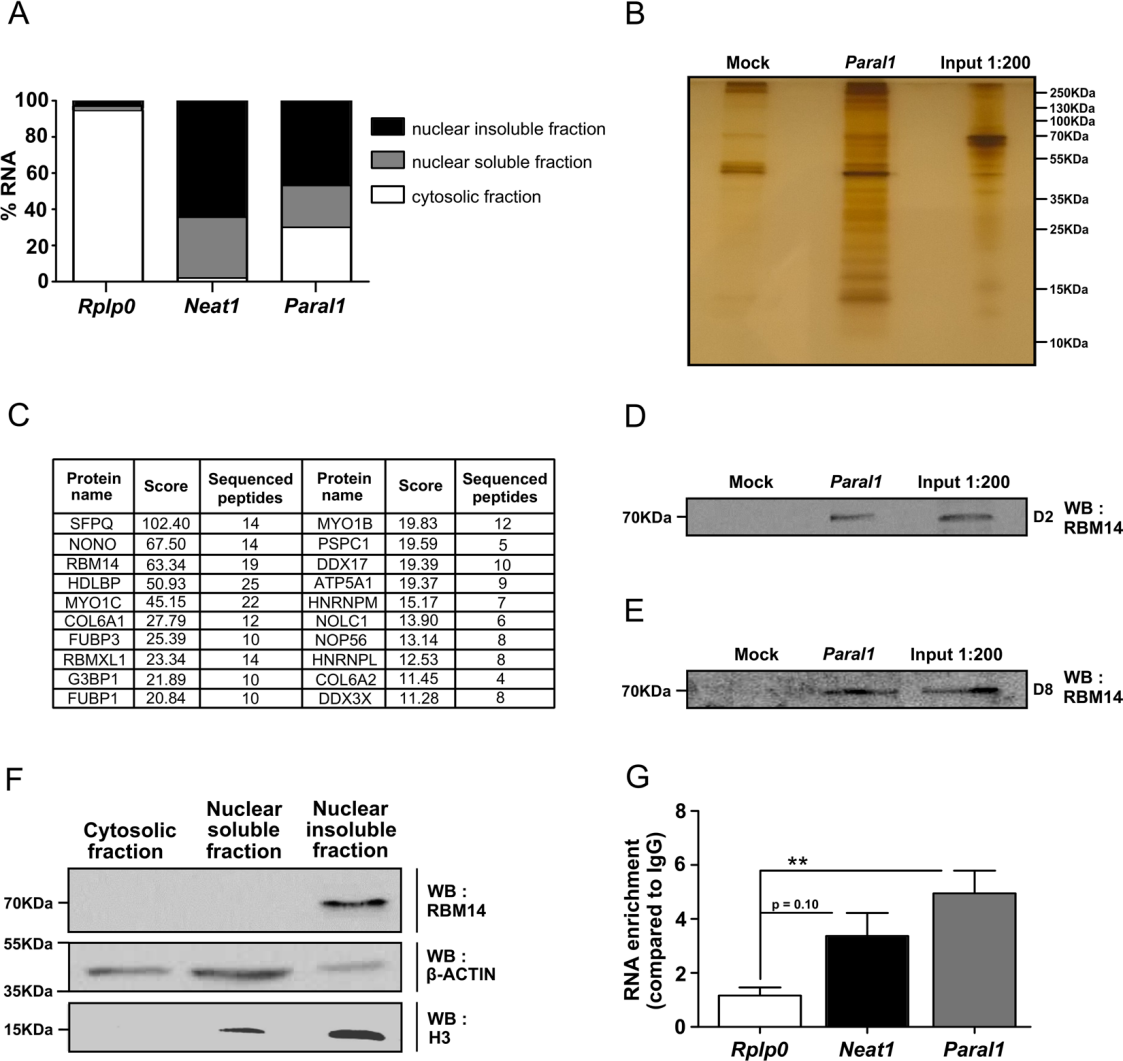
*Paral1* interacts with chromatin-bound RBM14. (**A**) Subcellular localization of *Paral1*. RNA from 3T3-L1 cells was fractionated into nuclear insoluble, nuclear soluble and cytosolic fractions at D5 and analysed for their content in *Paral1*, *Rplp0* and *Neat1*. *Rplp0* and *Neat1* RNA were used as cytosolic and nuclear RNA controls, respectively. (**B**) Proteins from RNA pull-down eluates (no RNA, *Paral1* and Input) were visualized by silver staining after SDS-polyacrylamide gel electrophoresis (PAGE). (**C**) Top hits of identified proteins by Q-Exactive nano-LC tandem mass spectrometry specifically interacting with *Paral1* in the RNA pulldown assay. (**D**) RNA pull-down eluates were analysed by western blotting for their content in RBM14 at D2. (**E**) RNA pull-down eluates were analysed by western blotting for their content in RBM14 at D8. (**F**) 3T3-L1 whole cell extracts (D5) were separated into nuclear insoluble, nuclear soluble and cytosolic fractions. Proteins were resolved by SDS-PAGE and identified by western blotting. β-actin and histone H3 were used as cytosolic and nuclear controls respectively. RNA pull-down eluates were analysed by western blotting for their content in RBM14. (**G**) RNA immunoprecipitation using an anti-RBM14 antibody. Total extracts were immunoprecipitated and *Paral1* level was measured by RT-qPCR experiments. *Rplp0* and *Neat1* RNA were used as a negative and positive control respectively.

As lincRNA functions are highly dependent on their subcellular localization^22^, we quantified *Paral1* transcripts in the chromatin (nuclear insoluble), nuclear (nuclear soluble) and cytosolic fractions from differentiated 3T3-L1 cells. Ribosomal *Rplp0* RNA was used as a cytosolic RNA control and *Neat1* as a chromatin-associated lincRNA^18^. *Paral1* was mainly nuclear (~70%) and predominantly detected in the chromatin fraction (~47%) (Fig. 3A). A RNA pull-down was performed using biotinylated *Paral1* as a bait (Fig. 3B). This assay identified by mass spectrometry (Fig. 3C) known components of paraspeckles [Splicing Factor Proline And Glutamine Rich (SFPQ), Non-POU Domain Containing, Octamer-Binding (NONO), Paraspeckle Component 1 (PSPC1) and RNA Binding Motif Protein 14 (RBM14)]^23^. Among them, RBM14/ Coactivator Activator (COAA) is not only an RNA-binding protein, but a secondary coactivator of several nuclear receptors^24,25^. The presence of RBM14 in RNA pulldown eluates from cellular extracts from differentiating (D2) and differentiated (D7) was validated by western blotting (Fig. 3D,E, Supplemental Fig. 11). Like *Paral1*, RBM14 was located in the chromatin fraction (Fig. 3F).

Immunoprecipitation using an antibody against RBM14 followed by RT-qPCR (RIP-qPCR) (Fig. 3G) showed that *Paral1* was specifically enriched (x5) in RBM14-containing complexes, confirming that RBM14 interacts specifically with *Paral1*. The *Rplp0* cytosolic RNA did not interact with RBM14, whereas the paraspeckle-associated *Neat1* lncRNA^16^ was enriched (x 3.4) in RBM14 immunoprecipitates. The interaction of RBM14 with *Paral1* was mapped to the 5′ half of *Paral1* (Supplemental Fig. 12), whose secondary structure prediction did not reveal any peculiar features (Supplemental Fig. 13).

### RBM14 cooperates with *Paral1* in regulation of adipocyte differentiation

**Figure 4:**
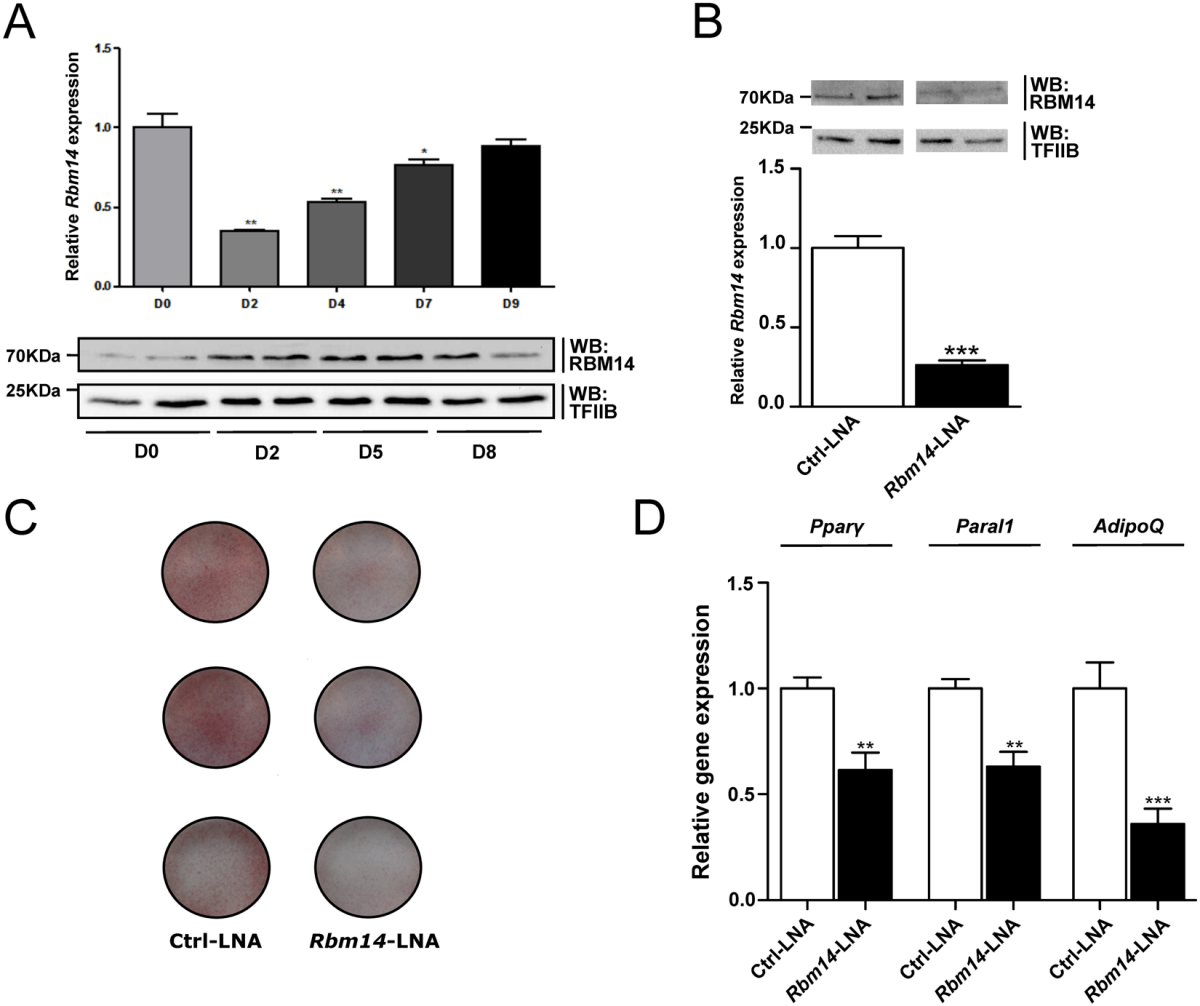
RBM14 protein expression increases during adipogenesis and is required for the adipogenic process. (**A**) RBM14 RNA level (upper panel) and protein level (lower panel) were assayed by RT-QPCR and western blotting respectively. RT-QPCR results were expressed as the mean ± S.E.M. (n = 3) relative to the indicated control in non-treated 3T3-L1 preadipocytes arbitrarily set to 1. Values were compared using ANOVA followed by a Tukey’s post hoc test. *p < 0.05; **p < 0.01. RBM14 protein was visualized in 3T3-L1 cells lysates (70 µg) at D0, D2, D5 and D8 by western blot using a specific anti-RBM14 polyclonal antibody. (**B**) *Rbm14* knockdown by LNA gapmer transfection. Control (Ctrl-LNA) or LNA gapmers targeting *Rbm14* (*Rbm14*-LNA, RBM14/2-LNA) were transfected at D0 and *Rbm14* RNA and protein levels were assayed by RT-qPCR and western blotting as above at D8. (**C**) ORO staining of 3T3-L1 cells at D8. (**D**) *AdipoQ* expression in RBM14-depleted cells. *Pparγ*, *Paral1* and *AdipoQ* transcript abundance was measured by RT-qPCR in RNA extracted from 3T3-L1 cells at D2. Results are expressed as the mean ± S.E.M. (n = 3) relative to the indicated control in non-treated 3T3-L1 preadipocytes arbitrarily set to 1. Values were compared using a t-test. *p < 0.05; **p < 0.01; ***p < 0.001.

RBM14 protein expression increases during 3T3-L1 differentiation similar to *Paral1* (Fig. 4A). *Rbm14* mRNA expression however did not strictly match protein expression (Fig. 4A), suggesting that post-transcriptional processes may influence RBM14 protein stability. We investigated whether RBM14 is also required for 3T3-L1 adipogenesis by loss-of-function experiments (Fig. 4B and Supplemental Fig. 14). RBM14-depleted 3T3-L1 cells accumulated less lipid at D8 (Fig. 4C), and *adiponectin* and *Pparγ* gene expression was decreased (Fig. 4D). *Paral1* and RBM14 are thus both necessary for adipocyte differentiation. A similar loss-of-function study was performed for the other *Paral1*-associated paraspeckles components (Supplemental Fig. 15). Both *Pspc1* and *Sfpq* knockdowns interfered with adipogenesis, whereas the contribution of NONO was not significant. Paral1 may thus belong to a large functional protein complex comprising several paraspeckle components.

### *Paral1* potentiates RBM14 coactivation of PPARγ transcriptional activity

**Figure 5:**
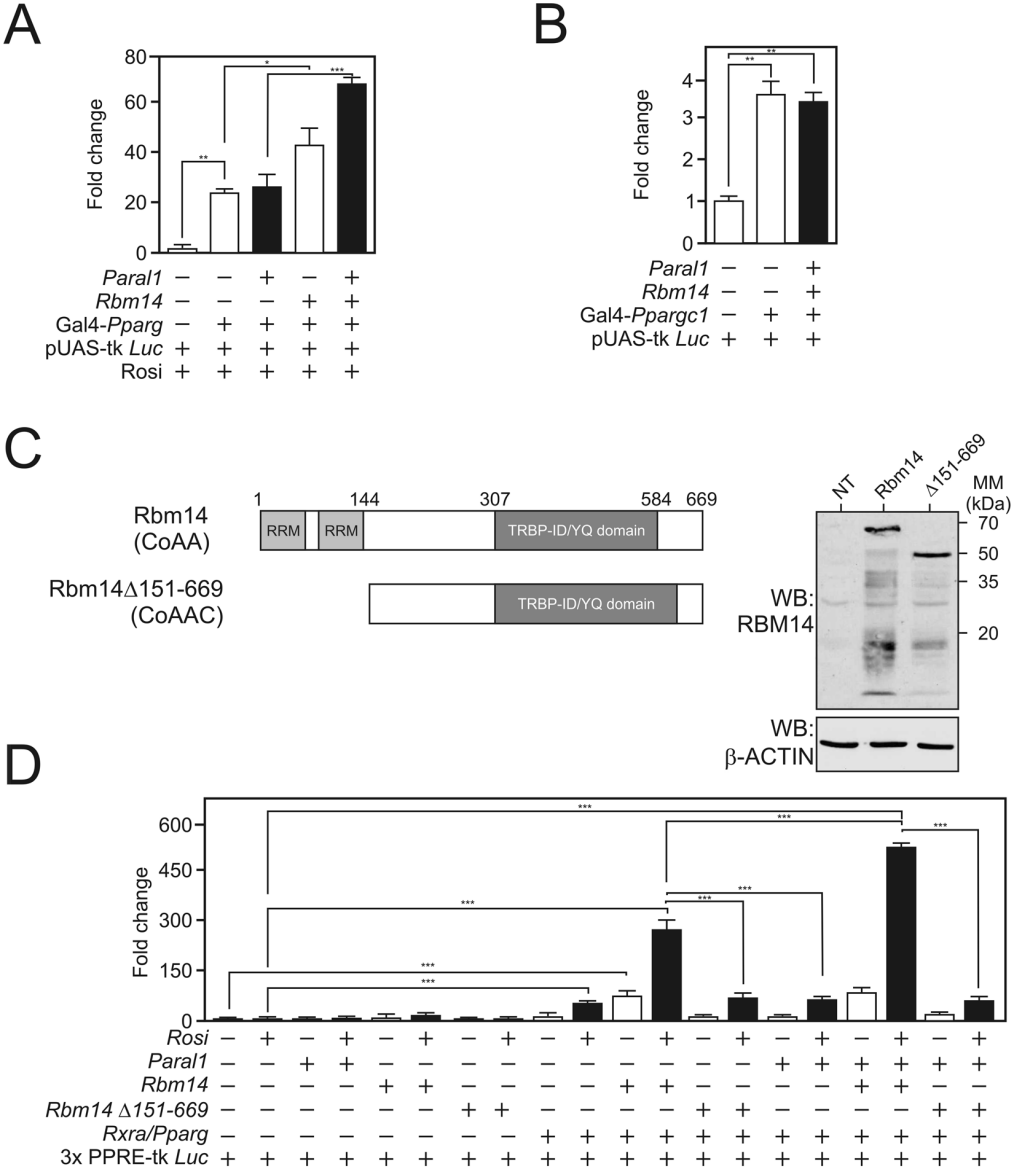
*Paral1* potentiates RBM14 coactivation of PPARγ. (**A**,**B**) One-hybrid transactivation assay monitoring the transcriptional potential of PPARγ (A) or PGC1α (**B**). HEK cells were transfected using the indicated combination of reporter (pUAS-tk Luc) and expression (pGal4-*Pparγ*, pcDNA3-*Rbm14*, pcDNA3-*Paral1*) vectors. Results are expressed as fold change relative to luciferase level detected in cells transfected without the indicated transcription factor. Results are expressed as means ± S.E.M (n = 3–5). The statistical significance of differences was analyzed by ANOVA followed by a Tukey’s post hoc test. *p < 0.05; **p < 0.01; ***p < 0.001. (**C**) Structure and expression of RMB14. The structure of wild type RBM14 is depicted with numbers indicating aminoacid sequence positions. Right panel: western blot analysis of RBM 14 derivatives when expressed in HEK cells (NT: non transfected). (**D**) PPRE-dependent transactivation assay. HEK cells were transfected with a PPRE-driven reporter vector and the indicated combination of expression vectors. Results are expressed as fold change relative to luciferase level detected in cells transfected without the indicated transcription factor. Results are expressed as means ± S.E.M (n = 3). The statistical significance of differences was analyzed by ANOVA followed by a Tukey’s post hoc test. *p < 0.05; **p < 0.01; ***p < 0.001.

As our data pointed to a regulatory role of the *Paral1*:RBM14 complex in PPARγ–driven events, we investigated its potential contribution to PPARγ transcriptional activity. Using a 1-hybrid assay in which Gal4 DBD-fused PPARγ activity was monitored in the presence or not of overexpressed *Paral1* and/or *Rbm14*, we observed that, in contrast to RBM14 which increased PPARγ transcriptional activity, *Paral1* had no effect on its own in this assay (Fig. 5A). However, *Paral1* potentiated RBM14 coactivation of PPARγ. This combination had no effect on PGC1α-regulated transactivation (Fig. 5B).

RBM14/NCoAA comprises 2 main functional domains including at its N-terminus 2 RNA recognition motifs (RRMs) and a large TRBP/AIB3 interacting domain (TRBP-ID), that mediates its interaction with nuclear receptor coactivators (Fig. 5C ^24^). We monitored PPARγ transcriptional activity using a PPARγ response element (PPRE)-driven reporter gene and an expression vector coding for RXRα, PPARγ’s obligate heterodimerization partner, in the presence of wild type RBM14 or of a RBM14 N-terminally truncated mutant (Fig. 5C,D). In this system, the RXRα/PPARγ dimer induced a 15-fold increase over control (no RXRα/PPARγ) of the reporter gene activity in the presence of rosiglitazone (Rosi), which was not altered by the overexpression of *Paral1*. In contrast, wild type RBM14 significantly increased both basal (FC = 15) and Rosi-induced (FC = 240) luciferase activity. The RBM14 deletion mutant was inactive in similar conditions. Overexpression of *Paral1* did not affect the ability of RBM14 to potentiate the basal activity level of the system, but dramatically increased its activity in the presence of rosiglitazone (FC = 33), confirming the functional synergy between *Paral1* and RBM14 on PPARγ-mediated transcription. In sharp contrast, the RRM-truncated RBM14 mutant was unable to convey such a potentiation. We also observed that a TRBP-ID deleted RBM14 was devoid of any activity in this system (data not shown). While requiring further investigation to reach a definitive conclusion, this suggests that the RBM14 RRM domain is required for such a functional interaction to occur.

### Adipose tissue inflammation lowers Paral1 expression in murine models of obesity

**Figure 6:**
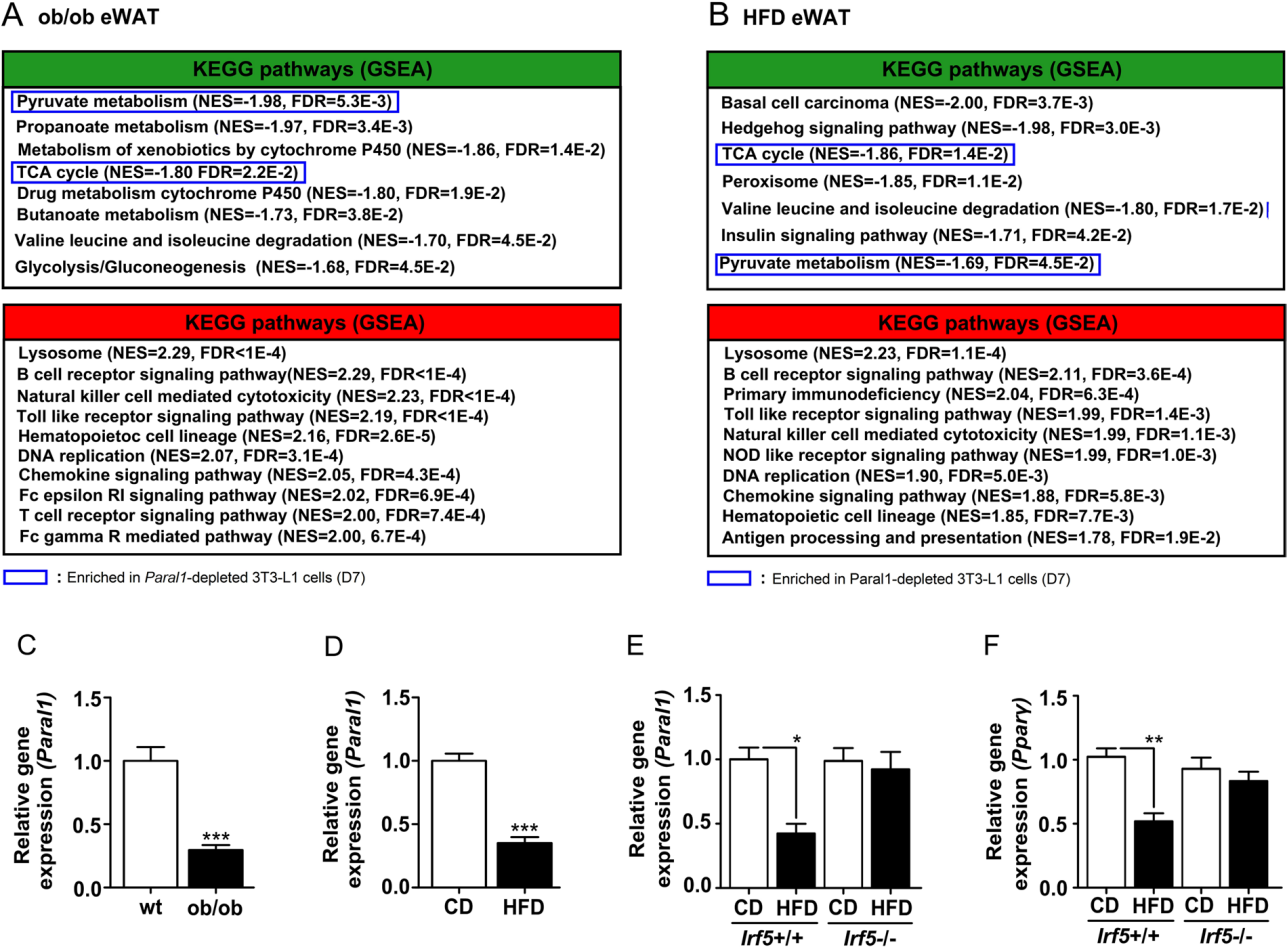
*Paral1* expression is decreased in obese eWAT in an *Irf5*-dependent manner. (**A**) GSEA was performed using the KEGG pathway gene sets. Red: up-regulated in obese (ob/ob) eWAT, green: down-regulated in obese (ob/ob) eWAT. (**B**) GSEA was performed using the KEGG pathway gene sets. Red: up-regulated in obese (HFD) eWAT, green: down-regulated in obese (HFD) eWAT. Framed: pathways common to both obese eWAT and *Paral1*-depleted 3T3-L1 cells at D7. NES: normalized enrichment score, FDR: false discovery rate. (C) *Paral1* expression in eWAT. eWAT RNA from wild type (wt) C57Bl6/J and from ob/ob mice were analysed for their content in *Paral1* transcripts by RT-qPCR. Results are expressed as the mean ± S.E.M. (n = 6–8) relative to the wild type level arbitrarily set to 1. Values were compared using a t-test. *p < 0.05; **p < 0.01; ***p < 0.001. (**D**) *Paral1* expression in eWAT. eWAT RNA from wild type (wt) C57Bl6/J fed either a chow diet (CD) or a high fat diet (HFD) were analysed for their content in *Paral1* transcripts by RT-qPCR. Results are expressed as the mean ± S.E.M. (n = 6–8) relative to the wild type level arbitrarily set to 1. Values were compared using a t-test. *p < 0.05; **p < 0.01; ***p < 0.001. (**E**) *Paral1* and *Pparγ* expression in eWAT. eWAT RNA from wild type (*Irf5* ^+/+^) or from *Irf5*-deficent-mice (*Irf5* ^−/−^) fed either a chow diet (CD) or a high fat diet (HFD) were analysed by RT-qPCR. Results are expressed as the mean ± S.E.M. (n = 5–8) relative to the wild type level arbitrarily set to 1. Values were compared using a t-test. * p < 0.05; ** p < 0.01; ***p < 0.001.

*Paral1* expression was therefore assessed in 2 mouse models of obesity. Epididymal (e)WAT from both leptin-deficient (*ob/ob*) and high-fat diet-fed (HFD) mice displayed clear signs of increased expression of inflammation-related pathways (Fig. 6A,B, Supplemental Fig. 16), in agreement with our previous studies^26^. GSEA comparing gene expression patterns of *Paral1*-depleted 3T3-L1 cells at D7 to that of *ob/ob* and HFD mouse eWAT revealed that pathways related to oxidative phosphorylation were commonly dysregulated (Fig. 6A,B and Supplemental Fig. 10). Epididymal WAT from obese mice displayed a markedly decreased expression of *Paral1*, in line with our data showing that depletion of *Paral1* yields dysfunctional 3T3-L1 cells, (Fig. 6C,D). PPARγ gene expression was also decreased in eWAT from obese mice, but not RBM14 (Supplemental Fig. 17A,C and B,D respectively). Increased pro-inflammatory cytokines production is a hallmark of obese WAT. TNF stimulation of differentiated 3T3-L1 cells, mimicking part of the M1 macrophage-induced inflammatory response, decreased *Paral1* expression, suggesting that obesity-induced inflammation may regulate *Paral1* expression (Supplemental Fig. 18). IRF5 is a key driver of the pro-inflammatory response in WAT and *Irf5* gene knockout protects from metabolic damages caused by diet-induced obesity, notably through impaired IL-1β and TNF release^27^. eWAT from HFD-fed *Irf5* ^+/+^ mice displayed decreased *Paral1* expression, which paralleled *Pparγ* expression, when compared to chow diet (CD) fed mice (Fig. 6E,F). In sharp contrast, eWAT from HFD fed *Irf5* ^−/−^ mice displayed *Paral1* and *Pparγ* expression levels comparable to CD-fed *Irf5* ^−/−^ mice (Fig. 6E,F). Thus *Paral1* expression is impacted by TNF *in vitro* and by a chronic pro-inflammatory background in an *Irf5*-dependent manner *in vivo*.

### Identification of a human *Paral1* homolog with decreased expression in obesity

**Figure 7:**
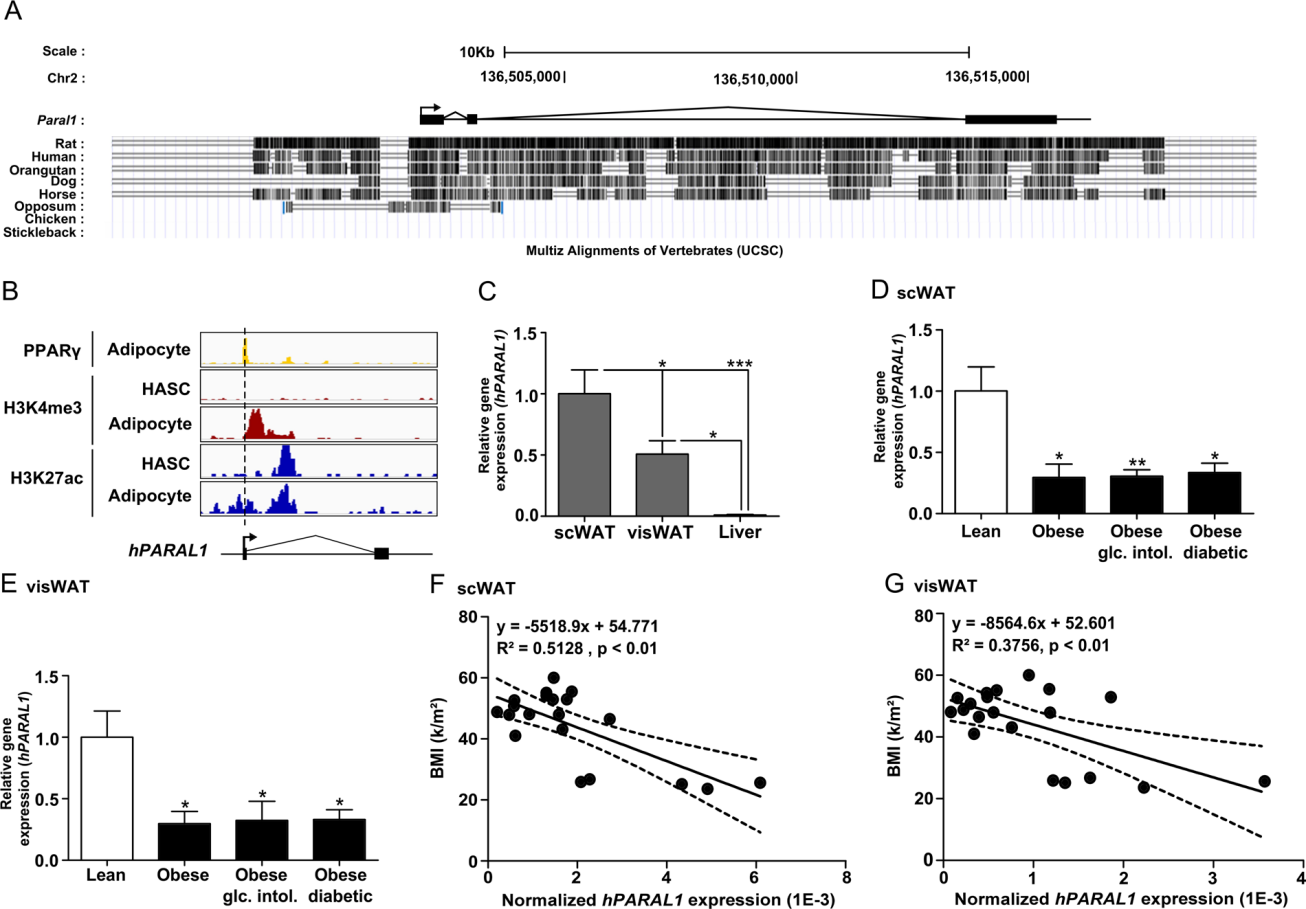
Identification of a human *Paral1* homolog. (**A**) Multiz alignments of vertebrate homologs to mouse *Paral1* in different species visualized with the UCSC Genome browser^59^. (**B**) Gene tracks visualized in IGV show the PPARγ ChIP-seq signal [from differentiated human adipose stem cells (HASC), yellow], the H3K4me3 ChIP-seq signal (from undifferentiated and differentiated HASC; same scale; red) and the H3K27ac ChIP-Seq signal (from undifferentiated and differentiated HASC; same scale; blue) at the human *PARAL1* locus. (**C**) *hParal1* expression level was measured by RT-qPCR in total subcutaneous adipose tissue (scWAT), omental adipose tissue (visWAT) and liver. Results are expressed as the mean ± S.E.M. (n = 6) relative to the wild type level arbitrarily set to 1. The statistical significance of differences were assessed by ANOVA and Tukey’s post hoc test. *p < 0.05; **p < 0.01; ***p < 0.001. (**D**) *hPARAL1* expression level was measured in subcutaneous adipose tissue (scWAT) by RT-qPCR. Results are expressed as the mean ± S.E.M. (n = 5) relative to lean control level arbitrarily set to 1 (not shown). The statistical significance of differences were assessed by ANOVA and Dunnett’s post hoc test. *p < 0.05; **p < 0.01; ***p < 0.001. (**E**) *hParal1* expression level was measured in visceral adipose tissue (visWAT) by RT-qPCR. Results are expressed as the mean ± S.E.M. (n = 5) relative to lean control level arbitrarily set to 1. The statistical significance of differences was assessed by ANOVA and Dunnett’s post hoc test. *p < 0.05; **p < 0.01; ***p < 0.001. (**F**) Correlation between *hPARAL1* expression level in scWAT and BMI (n = 20). G) Correlation between *hParal1* expression level in visWAT and BMI (n = 20).

The most recent compendium of human lncRNAs^28^ suggest that functional lncRNAs are most conserved across species. Sequence alignment across multiple species showed that mouse *Paral1* displays significant similarities in the transcribed region with a human homolog (Fig. 7A). Identified as ENSG00000243961.2 in the human lncRNA repertoire (http://fantom.gsc.riken.jp/cat/v1/#/genes/ENSG00000243961.2), *hPARAL1* is also flanked by *PAK7* and *ANKRD5* and displays a conserved promoter, indicating a potential functionality^28^. Like its murine counterpart, it harbors no significant coding potential and is induced during adipocyte differentiation^28^, a feature confirmed by the analysis of epigenetic marks around the TSS in undifferentiated and differentiated human adipose-derived stem cells [hASC^21^], (Fig. 7B). Expression atlas data (Supplemental Fig. 19) and RT-qPCR assays (Fig. 7C) show that *hPARAL1* expression is highest in lung and breast and significantly detected in subcutaneous (sc) and omental (vis)WAT.

Quantification of *hPARAL1* transcripts in subcutaneous (sc)WAT and visceral (vis)WAT from lean, obese, glucose-intolerant obese and obese diabetic patients showed decreased *hPARAL1* expression in obesity (Fig. 7D,E). In line with mouse data, altered pathways in human obese visWAT again pertained to oxidative phosphorylation and the PPAR signaling pathway in a pro-inflammatory context, both being biological processes affected upon *Paral1* depletion (Supplemental Fig. 20). *hPARAL*1 expression was inversely correlated to BMI (Fig. 7F,G) but not to other biometric or biochemical parameters (Supplemental Table 6), suggesting an exclusive link between h*PARAL1* expression and WAT dysfunctions in human WAT.

## Discussion

**Figure 8:**
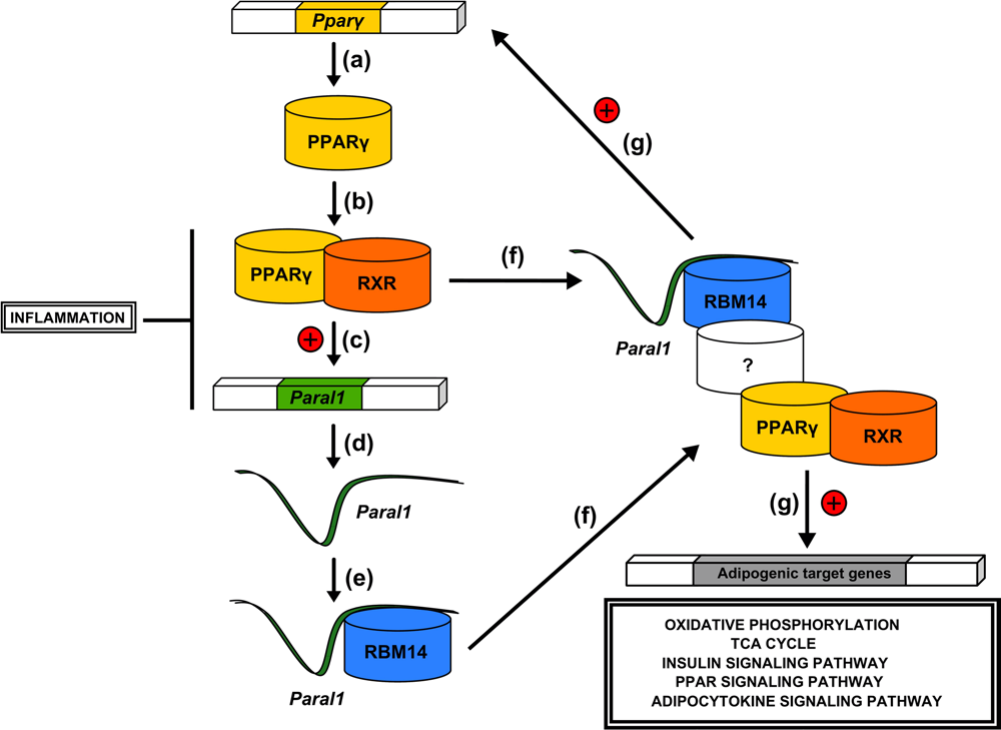
*Paral1* mechanism of action. PPARγ expression is activated during adipogenesis (**a**) creating an heterodimer complex with RXR (**b**) in order to regulate adipogenic factors such as *Paral1* (**c**) necessary for adipogenesis. The *Paral1* RNA transcript (**d**) interacts with RBM14 (**e**) potentiating its coactivating function (**f**). The interaction between RBM14-Paral1 and PPARγ-RXR complexes (**f**) promotes PPARγ activity leading to positive feedback in the upregulation of adipogenic factors (**g**).

Adipocyte differentiation and function relies on an intricate network of interconnected transcription factors centered on PPARγ^29^. We report here the identification and characterization of the lincRNA *Paral1* as a novel member of this adipogenic master regulatory network. *Paral1*, whose expression is a feature of mature adipocytes, is required for PPARγ expression and activity during adipogenesis and sustains adipocyte functions. In line, an unbiased search for potential functions of *Paral1* based on tissue specific co-expression studies^30^ predicted a role for *hPARAL1* in triglyceride and pyruvate metabolisms. Interestingly, *Paral1* expression is decreased in an obesogenic context in both humans and mice and is sensitive to pro-inflammatory signals suggesting that loss of *Paral1* expression may contribute to WAT dysfunction in obese individuals with chronic inflammation. Our data also show that *Paral1* expression parallels that of PPARγ, as both transcripts increase during adipocyte differentiation. The occurrence of a PPARγ binding site upstream of the *Paral1* TSS suggests that PPARγ may drive the expression of a coactivating RNA molecule. This would establish a positive feedforward loop on PPARγ expression (Fig. 8), raising the question of the impact of PPARγ agonism on *Paral1* expression. In mature 3T3-L1 adipocytes, acute treatment by insulin-sensitizing thiazolidinediones (TZD such as rosiglitazone or pioglitazone) increases the expression of many genes induced during differentiation, with a few notable exceptions including PPARγ itself whose expression is decreased upon TZD treatment^31^. We could replicate this finding and, as expected from our data, *Paral1* expression followed that of PPARγ (data not shown). While the mechanistic basis of this repression is still elusive^31,32^, this demonstrates that *Paral1* expression is exquisitely dependent on PPARγ expression level, a feature that we also confirmed in WAT from ob/ob mice treated for 5 days by rosiglitazone (3mpk/day,^26^). In this setting, TZD treatment increased PPARγ expression and that of several bona-fide target genes such as adiponectin, and also induced *Paral1* expression (data not shown and^26^).

The molecular basis of *Paral1* pro-adipogenic activity stems from its ability to interact with and potentiates the co-activating potential of RBM14, whose expression parallels that of *Paral1* during adipocyte differentiation. This newly identified property of RBM14 is probably due to its ability to act as an indirect coactivator, through synergistic interactions with nuclear receptor coactivators (hence its alias CoActivator Activator CoAA)^24,33^. Our work extends its role, initially reported for estrogen and glucocorticoid receptor-dependent transcriptional activation^34^, to the pro-adipogenic nuclear receptor PPARγ. RBM14, a hnRNP-like protein, encompasses 2 RNA binding modules (RRM), which are likely to interact with RNA molecules such as *Paral1*, as described for hnRNP U and Xist^35^. In the chimeric transcription assay used here, we could indeed show that *Paral1* function is dependent on the RRM domains.

We also note that RBM14/CoAA inhibits the transcriptional activity of the osteoblastic Runx2 transcription factor, a differentiation pathway that opposes commitment to the adipocyte lineage^36^. Together with other studies^37^, our work indicates that lincRNAs act as regulators of expression/activities of key developmental transcription factors. Another way by which lncRNAs regulate gene expression and ensuing biological processes is to act in cis at neighbouring protein-coding loci^38^. *Paral1* is flanked by *Ankrd5* and *Pak7*, none of these genes being reportedly involved in adipogenesis and dysregulated upon *Paral1* expression modulation, thereby excluding a possible contribution of *Paral1* through such a mechanism.

As noted above, RBM14 possesses RNA binding activity and is a component of paraspeckles, which assemble on the lincRNA *Neat1*. Interestingly, we found that *Paral1* also interacts with paraspeckle components NONO, PSPC1 and SFPQ which are three multifunctional nuclear factors and bind *Neat1*. This suggested that RBM14-*Paral1* could be involved in splicing events. However, our exon microarray analysis did not reveal major changes in the splicing of adipogenic genes (data not shown). This however does not rule out other paraspeckle-associated roles for this RNA-protein complex which remain to be formally investigated, such as the nuclear retention of edited RNA molecules known to play a role in cellular differentiation^39^.

About 30% of total *Paral1* is localized in the cytosol. Although our approach did not allow to appreciate potential roles of *Paral1* as a miRNA sponge, we note that *Paral1* could hybridize to mir27a, a post-transcriptional regulator of PPARγ^40^ through its sequence ACUGUGA.

*Paral1* interacts also with Eukaryotic Translation Elongation Factor 1 Alpha 1 (EEF1A1) and with Tubulin beta (Tubb), a component of the centriole cytoskeleton with which RBM14 interacts^41^, hinting at a possible role in mRNA translation or cell division. The basis for this subcellular repartition is unknown. A recent report demonstrated that PSPC1 is involved in the nuclear export of adipogenic RNAs, including PPARγ^42^. These observations raise the possibility that *Paral1* may be part of this RNA shuttling complex, since the RNA shuttling protein DDX3X interacts with *Paral1* (Fig. 3C) and PSPC1^42^.

In conclusion, we have identified on the basis of an unbiased genome-wide approach a novel lincRNA with functions in adipocyte physiology, whose expression is decreased in obese rodents and humans. At least part of its mechanism of action can be attributed to co-activating properties of PPARγ, thereby impacting on genes involved in metabolic regulations. This adds a new piece to the puzzle of lncRNA contribution to adipogenesis. In this context, the structural versatility of RNA molecules make them attractive druggable entities^43^ and a complete inventory of anti- or pro-adipogenic lncRNAs may expand the therapeutic repertoire to combat obesity.

## Experimental procedures

### Chemicals

Dulbecco’s Modified Eagle’s Medium, alpha Modified Eagle’s Medium, Cosmic Calf Serum and Fetal Bovine Serum (FBS) were from Life Technologies. GW4064 was from Tocris. Ascorbic acid, β-glycerophosphate, bovine insulin, IBMX, dexamethasone, indomethacine, pioglitazone and rosiglitazone were from Sigma.

### Cell culture

3T3-L1 and 3T3-442A cells were routinely grown and differentiated as described^26^. Mesenchymal stem cells (MSC) were isolated from adult mouse bone marrow and maintained in αMEM (Life Technologies) as described^44^.

### Oil Red O staining

Oil red O (ORO) staining was performed at D8 as described^8^.

### Triglycerides quantification

Triglycerides quantification were performed at D8 as described^45^.

### RNA extraction and RT-qPCR

Total RNA was extracted, analyzed by RT-qPCR and transcript level were quantified as described^26^.

### Microarray analysis

Total RNA (100–300ng) from 3T3-L1 cells was processed for labelling, purification and hybridized to Affymetrix Genechip Mouse Genome 430 2.0 or to Mouse Transcriptome Array 1.0 according to the manufacturer’s protocol. Raw data (available on the GEO website under the accession number GSE 97241) were pre-processed using the GCCN and SST algorithms (Expression Console, v1.4.1. Affymetrix). RMA background correction and gene-level probe set summarization were performed with the Partek Genomics Suite software (v6.6, Partek Inc.). Microarray analysis of leptin-deficient (ob/ob) mouse WAT has been described elsewhere^26^. Microarray data from mouse WAT fed either a chow or a high fat diet were from the GEO dataset GSE21069 ^46^. Human WAT RNAs were analysed as described^26^.

Human gene symbols were attributed to each murine gene using the Orthologue Conversion software (https://biodbnet-abcc.ncifcrf.gov/db/dbOrtho.php)^47^ and resulting files were analysed with the GSEA software [Broad Institute, v2.2.2^48^]. Pathway-enrichment scores were calculated with the GSEA pre-ranking tool and the KEGG Pathway gene set. Default parameters were used except for the permutation number (10,000) and the enrichment score statistic (weighted). The GEO dataset is available under the number GSE97241.

### Data mining and bioinformatics

#### Long non-coding RNA identification

Murine lncRNA sequences were extracted from the NONCODE database v.3 (http://www.noncode.org/NONCODERv3/; mouse genome reference: mm9).

Gene Expression Omnibus (GEO) datasets were: GSE84888 for differentiating human adipose stromal (hASC) and murine 3T3-L1 cells^21^ and GSE92590 for isolated primary mouse adipocytes from WAT^49^. RNA-Seq data were from the GEO dataset GSE35724^50^.

ChIP-Seq data were analyzed using the Galaxy Cistrome platform^11^. RNA-Seq and ChIP-Seq data were visualized using the Integrative Genomics Viewer software (IGV, v2.3.14, Broad Institute).

#### Coding potential analysis and proteomic databases

Three softwares were used with default parameters to determine lincRNAs’ coding potential (CPC, http://cpc.cbi.pku.edu.cn/)^51^; CPAT, http://lilab.research.bcm.edu/cpat/index.php)^52^ and GenView2 (http://bioinfo.itb.cnr.it/~webgene/wwwgene.html). GAPDH and XIST RNA were used as representative of mRNA and lincRNA respectively. Potential ORFs were converted into protein sequences and matching peptides were searched in four protein databases [UniProtKB (SwissProt and TEMBL; http://www.uniprot.org/); PDB (http://www.rcsb.org/pdb/home/home.do); Ensembl (www.ensembl.org/)].

### Total protein extraction, western blotting and antibodies

Proteins (30–100 µg) were extracted and analysed by western blotting as described^53^. Primary antibodies used in this study were: anti-RBM14 (Euromedex, ref. 10196-1-AP), anti-PPARγ (Santa Cruz, ref. sc-7196), anti-TFIIB (Santa Cruz, ref. sc-225), anti-β-actin (Santa Cruz, ref. sc-1616), anti-H3 (Abcam, ref. ab1791) and control IgG (Merck-Millipore, ref. 17–658). Secondary antibodies were anti-rabbit IgG-peroxidase antibody (Sigma, ref. A0545) or anti-goat IgG-peroxidase antibody (Sigma, ref. A5420).

### Subcellular fractionation

3T3-L1 cells were plated in P100 dishes and differentiated as described above. At indicated times, cells were washed twice using ice-cold 1x PBS and lysed in 500 µL lysis Buffer 1 [10 mM HEPES pH 7.9, 10 mM KCl, 1.5 mM MgCl_2_, 0.34 M sucrose, 10% glycerol, 40 U/mL RNasin and protease inhibitors (Roche)] supplemented with 0.1% Triton-X100 and 1 mM DTT upon use. Lysates were centrifuged to separate cytosolic and nuclear fractions (1,300 G, 5′, 4 °C). After removal of the upper lipid phase, supernatants (cytosolic fraction) were centrifuged (16,000 G, 5′, 4 °C) and used for RNA and protein characterization. Nuclei were washed once into 400 µL lysis buffer 1 supplemented with 1 mM DTT extemporarily and incubated in 100 µL lysis buffer 2 (10 mM HEPES pH 7.9, 3 mM EDTA pH8, 0.2 mM EGTA pH8, 40 U/mL RNasin, 1 mM DTT and protease inhibitors) for 30 min. on ice. Insoluble and soluble fractions were obtained by centrifugation (1,300 G, 5′, 4 °C). The insoluble fraction was digested with benzonase [50 mM Tris-HCl, pH7.5, 1 mM MgCl_2_, 3 mM EDTA pH8, 0.2 mM EGTA pH8 supplemented with 25U benzonase (Millipore) and 1 mM DTT extemporarily] for 20 min. on ice.

### Oligonucleotide design and synthesis

LNA gapmers complementary to mouse *Rbm14* mRNA (*Rbm14*-LNA) were designed using the online Exiqon software (https://www.exiqon.com/ls/Pages/GDTSequenceInput.aspx): *Rbm14*-LNA: ATGACTGAGTGCGGTA; *Paral1*-LNA: AGGAGCATAATGAATA. Exiqon LNA gapmers were synthesized by Exiqon (Qiagen) with a phosphorothioate backbone and purified by a standard desalting method.

### Plasmids

The pcDNA3-Myc-*Rbm14* vector and derivatives were from D. Monté (Univ. Lille, France)^54^. Other plasmids used in this study were the pUAS-tk-Luc vector^55^, pSG5-hRXRα^55^, pcDNA3-Flag-*Pparγ* ^56^, pGal4-*PGC1α* vector^57^. pGal4-empty vectors was from Addgene. The pGL4.25 vector was from Promega. The pcDNA3.1 vector was from Life Technologies.

The *Paral1* RNA was reverse-transcribed and amplified by PCR with primers containing BamHI and NotI restriction sites (Supplemental Table 1) then ligated into the pCR blunt II TOPO vector (TOPO TA Cloning Kit, Life Technologies). The cDNA insert was then excised as a BamHI/ fragment and inserted into pcDNA3.1 to generate the pcDNA3-*Paral1* plasmid.

pRetroX-*Paral1* and pRetroX-*Pparγ* vectors were generated by ligating inserts into the pRetrox-Tight-Pur plasmid (ClonTech) as BamHI/NotI or NotI/XbaI inserts.

The pGL4-3xPPRE Luc reporter vector was generated by inserting an oligonucleotide containing 3 consensus PPRE sequences (in bold) (5′-AAGCTTGACAGGGGACC**AGGACAAAGGTCA**CGTTCGGGAAGCTTGTCGACAGGGGACC**AGGACAAAGGTCA**CGTTCGGGAAGCTTG TCGACAGGGGACC**AGGACAAAGGTCA**CGTTCGGGAAGCTT-3′) into the pGL4.25 [luc2CP/minP] backbone (Promega).

### Biotinylated RNA synthesis and purification

pcDNA3-*Paral1* was linearized by NotI, and biotinylated RNA transcripts were synthesized with the MEGAscript® T7 Transcription Kit (Ambion) and biotin-CTP (Enzo-Life Sciences). RNA was purified as suggested by the manufacturer (Ambion). RNA integrity and biotinylation efficiency were checked by agarose gel analysis and dot blotting, respectively.

### RNA pulldown assay

3T3-L1 cell lysates were pre-cleared [1 mg protein for 40 µL streptavidin-coupled Dynabeads M-280 (Invitrogen)] for 3 hours. Beads were removed by magnetic separation. Biotinylated *Paral1* RNA was incubated at 65 °C for 5 minutes then at room temperature for 10 min. *Paral1* RNA (60 µg) was coupled to streptavidin-coupled Dynabeads M-280 (1 µL/µg RNA) in water containing RNasin (40 U/mL) for 30 min. at 20 °C. The supernatant was removed by magnetic separation. Pre-cleared 3T3-L1 cell lysates (1 mg total protein) were incubated with RNA-coupled beads for 60 min. After 3 washes with washing buffer (25 mM Tris-HCl pH 7.4, 150 mM NaCl, 1 mM EDTA, 5% glycerol, 40 U/mL and protease inhibitors), beads were incubated in 100 µL 1.5x Laemmli buffer and supernatants were processed for further analysis.

### RNA immunoprecipitation

3T3-L1 cells were washed in ice-cold 1x PBS and incubated in 1x PBS-1% formaldehyde for 10 min. The crosslinking reaction was quenched with 0.25 M glycine for 5 min. Cells were washed once with 1x PBS and lysed in 25 mM Tris-HCl pH 7.4, 500 mM NaCl, 1 mM EDTA, 40 U/mL RNasin, 5% glycerol and protease inhibitors. Lysates were sonicated using a Bioruptor UCD-200 and centrifuged (16,000 G, 10 min. at 4 °C).Supernatants were brought to 150 mM NaCl using 25 mM Tris-HCl pH 7.4, 1 mM, 40 U/mL RNasin, 5% glycerol and protease inhibitors. Protein concentration was measured using the DC Protein Assay (Biorad).

Crosslinked lysates (1 mg) were incubated with 5 µL Protein G-coupled Dynabeads® (Invitrogen) for 1 hour at 4 °C. Beads were removed by magnetic separation and 1 µg of primary antibody against RBM14 or control IgG were added and incubated overnight. Magnetic Protein G-Dynabeads were coated overnight in 5% BSA, 100 µg/mL yeast tRNA and 40 U/mL RNasin, added to the pre-cleared lysate/antibody mix and incubated for 3 hours at 4 °C. Dynabeads were washed three times with Washing Buffer and incubated with a Reverse-Crosslinking Solution (100 mM Tris-HCl pH 7.5, 5 mM EDTA, 1% SDS, 10 mM DTT and 40 U/mL RNasin) during 15 min. at room temperature. Supernatants were digested with 20 µg proteinase K and incubated at 65 °C for 120 min. Immunoprecipitated RNAs were extracted with TRIzol according to the manufacturer’s instructions.

### siRNA transfection

3T3-L1 cells were transfected by siRNAs (100 nM) using INTERFERin (Polyplus Transfection) as described^26^. siRNAs were: control siRNA (Dharmacon, ON-TARGETplus non-targeting, ref. D-001810-10-10,), *Pparγ*-siRNA (Dharmacon, ON-TARGETplus, ref. L-040712-00-0010n), *Paral1/1*-siRNA (Silencer Select ref. 101240, Ambion) or *Paral1/2*-siRNA (Silencer Select ref. 101167, Ambion).

### Transient transfections and reporter gene assay

HEK293T cells (7.5 × 10^4^) were plated 1 day prior to transfection in 6-well plates. Plasmids were transfected using jetPEI according to the manufacturer’s instructions. A combination of the following vectors was used as indicated in the figure legends: pUAS-tk-Luc (1 µg), pGL4-3PPRE (1 µg), Gal4-*Pparγ* (100ng), Gal4-*PGC1α* (100ng), pcDNA3-Myc-*Rbm14* (250ng), pcDNA3-*Paral1* (1 µg), pcDNA3-Flag-*Pparγ* (500 pg) and pSG5-*RXRα Rxrα* (500 pg). The next day, HEK293T cells were trypsinized and transferred into 96-wells plates. Twenty four hours later, cells were incubated with either vehicle (DMSO) or indicated compounds [Rosiglitazone (2 µM), GW4064 (1 µM)] overnight. Cells were lysed and luciferase activities were measured as described^53^.

### Retroviral infection and clone selection

Phoenix cells (10^6^) were transfected using Lipofectamine 2000 (Life Technologies) and indicated plasmids according to the manufacturer’s instructions. Transfected plasmids were the pRetroX-Tight-Pur plasmids (pRetroX-empty, pRetroX-*Pparγ* or pRetrox-*Paral1*) and the pRetroX-Tet-On vector. After a 5-hour incubation in serum-free medium, cells were washed twice with 1x PBS and grown in complete medium supplemented with 10% FBS. Retroviral supernatants were collected and stored at −80 °C until use.

Stable cell line production: 3T3-L1 cells (2.5 × 10^4^) were plated into 12-wells plates and transduced overnight with the above retroviral supernatants (500 µL pRetroX-Tet-On supernatant and 500 µL pRetroX-Tight-Pur). Clones were selected with 10 µg/mL puromycin and 800 µg/mL G418 for 7-10 days. Doxycycline (5 µg/mL) was used as the Tet-ON system inducer.

### Electrophoresis, gel staining and mass spectrometry

Protein separation, in-gel trypsin cleavage and mass spectrometry analysis were carried out as described^58^. Peptide separation was performed using an EASY-nLC 1000 UHPLC (Thermo Scientific) equipped with a 75 µmX 2 cm Acclaim PepMap 100 pre-column with nanoViper fittings and a 50 µm I.D. × 500mm Acclaim PepMap RSLC analytical column (Thermo Scientific). Peptides were eluted using a 5%-30% acetonitrile gradient for 60 min. at a flow rate of 300 nL/min. The Q-Exactive instrument acquisition mode was set to the top 10 MS2 method. The survey scans were taken at 70,000FWHM (at m/z 400) resolving power in positive mode and using a target of 1e6 and default charge state of + 2. Unassigned and + 1 charge states were rejected, and dynamic exclusion was enabled for 30 sec. The scan range was set to m/z 300–1600 m/z. For MS/MS, a microscan was obtained at 17,500FWHM and with an isolation window of 3.0 m/z, using a scan range between m/z 200–2000 m/z. Tandem mass spectra were processed with the Thermo Scientific Proteome Discoverer software v 1.3. Spectra were searched against UniprotKB/Swiss-Prot mouse databases (version 09/2015) using the SEQUEST HT algorithm (v1.3.0.339). The search was performed choosing trypsin as the cleaving enzyme with one missed cleavage site allowed. Precursor mass tolerance was 10 ppm, and fragment mass tolerance was 0.1 Da. N-terminal acetylation, cysteine carbamidomethylation and methionine oxidation were set as variable modifications. Peptide identification was performed with the Percolator algorithm by selecting only peptides with a q-value < 0.01, which corresponds to a false discovery rate (FDR) of 1%.

### Animal experimentation

All animal experiments were approved by the ethical committee for animal experimentation of Institut Pasteur de Lille, Pierre and Marie Curie University and French Research Council guidelines. All methods were performed in accordance with the relevant French and European guidelines and regulations. Detailed procedures can be found elsewhere^8,27^.

### Human biopsies

Human tissue samples were provided by Dr P. Gélé and the Centre d’Investigations Cliniques (C.H.R.U. Lille, France). Human WAT samples were collected from patients undergoing abdominal surgery by laparoscopy or coelioscopy after informed consent was obtained. All procedures were approved by the C.H.R.U. Lille Ethical committee and were compliant to the French National Ethics Committee guidelines. Tissue samples from female patients (35–59 year-old) and are from the ABOS cohort (ClinicalTrials.gov identifier: NCT01129297). More details can be found elsewhere^26^.

### Statistical analysis

Statistical analysis was performed using Prism 6.0 (GraphPad Software, La Jolla, CA). Values are expressed as the mean +/− SEM. Statistical significance was evaluated using either a two-tailed t test or by one-way ANOVA followed by Tukey’s or Dunnett’s post hoc tests. *p < 0.05; **p < 0.01; ***p < 0.001.

### Data availability

The datasets generated during and/or analyzed during the current study are available from the corresponding author on reasonable request. Affymetrix raw files are available on the GEO web site.

## Electronic supplementary material

Supplemental information

Supplemental Table 1

Supplemental Table 2

Supplemental Table 3

Supplemental Table 4

Supplemental Table 5

Supplemental Table 6
