# Supplementary material for: The RBM14/CoAA-interacting, long intergenic non-coding RNA Paral1 regulates adipogenesis and coactivates the nuclear receptor PPARγ
Source: Sci Rep. 2017 Oct 26;7:14087. doi: 10.1038/s41598-017-14570-y (PMC5658386; doi:10.1038/s41598-017-14570-y)

SUPPLEMENTAL INFORMATIONS TO:

The RBM14/CoAA-interacting, long intergenic non-coding RNA Paral1 regulates adipogenesis and coactivates the nuclear receptor PPARγ.

François F. Firmin[1], Frederik Oger[1], Céline Gheeraert[1], Julie Dubois-Chevalier[1], Anne-Sophie Vercoutter[2], Fawaz Alzaid[3], Claire Mazuy[1], Hélène Dehondt[1], Jeremy Alexandre[1], Bruno Derudas[1], Quentin Dhalluin[1], Maheul Ploton[1], Alexandre Berthier[1], Eloise Woitrain[1], Tony Lefebvre[2], Nicolas Venteclef[3], François Pattou[4], Bart Staels[1], Jérôme Eeckhoute[1,*] and Philippe Lefebvre[1,*]

Supplemental Tables:

Supplemental Table 1. List of primers.

Supplemental Table 2. List of lincRNA with histone marks. Mouse lincRNA from the NONCODE database containing H3K4me3, H3K27ac or PPARγ marks in the vicinity of their TSS are listed and were used to generate the Venn diagram shown in Figure 1A.

Supplemental Table 3. Coding potential of *Paral1*. Coding potential of *Paral1* and of representative coding (*Gapdh*) and non-coding (*Xist*) RNA transcripts were obtained using CPC, Genview2 and CPAT softwares. Potential peptides were searched in protein databases (SwissProt, TEMBl, PDB, Ensembl).

Supplemental Table 4. Expression of white and brown adipose tissue gene markers in *Paral1*-depleted 3T3-L1 cells. Data were extracted after analysis by DNA microarray

Supplemental Table 5. List of *Paral1*-sensitive transcripts. A list showing the 310 down-regulated and the 210 up-regulated genes upon *Paral1* depletion is shown.

Supplemental Table 6. Correlation study between *hPARAL1* expression in human visWAT and scWAT, biometric or biochemical parameters. Correlation tests were performed using *hPARAL1* expression level as determined by RT-qPCR and clinical data. R^2 and p values as well as the resulting curve equation were determined using Graphpad Prism v6.0.

Supplemental Figures:

Supplemental Figure 1. Splicing variants of *Paral1* in 3T3-L1 cells. Primers specific for each exon or for a combination of exons were used in RT-qPCR assays. Results are expressed as the transcript level normalized to the reference *Rplp0* housekeeping gene expression level.

Supplemental Figure 2. ChIP-seq profiles at the *Pak7/Paral1/Ankrd5* locus. A) PPARγ, H3K4me3 and H3K27ac enriched sequences in 3T3-L1 cell chromatin were visualized using the IGV browser, as well as the RNA-seq profile. B) H3K4me3- and H3K27ac-enriched sequences in primary adipocyte and non-adipocyte fractions were visualized using the IGV browser.

Supplemental Figure 3. *Paral1* expression in differentiating 3T3-F442A cells. *Paral1* expression was monitored by RT-qPCR and normalized against the reference *Rplp0* housekeeping gene expression level. Results are expressed relative to the *Paral1* RNA level in preadipocytes arbitrarily set to 1.

Supplemental Figure 4. Osteogenic and adipogenic marker expression in mesenchymal stem cells. *Paral1* expression was monitored by RT-qPCR and normalized against the reference *Rplp0* housekeeping gene expression level. Results are expressed relative to the *Paral1* RNA level in undifferentiated MSC arbitrarily set to 1.

Supplemental Figure 5. Adipocyte-specific genes are downregulated in *Paral1*-depleted cells. Gene expression after Ctrl-siRNA or *Paral1/1*-siRNA transfection of 3T3-L1 cells at D0 was assessed by RT-qPCR at D2. Results are expressed as the mean ± S.E.M. (n=3) relative to the *Paral1* RNA level in Ctrl-siRNA transfected preadipocytes arbitrarily set to 1. Values were compared using a t-test. * $p < 0.05$; ** $p < 0.01$; *** $p < 0.001$.

Supplemental Figure 6. ORO staining of transfected 3T3-L1 cells. 3T3-L1 cells were transfected or not (NT) at D2 with Ctrl-siRNA, *Pparγ*-siRNA, *Paral1/1*-siRNA or *Paral1/2*-siRNA. Cells were fixed and stained by Oil Red O at D7. Three independent experiments are shown from top to bottom.

Supplemental Figure 7. *Ankrd5* knockdown does not affect adipogenesis. Control (Ctrl-siRNA) or siRNA targeting *Ankrd5* were transfected at D0 in 3T3-L1 cells and lipid accumulation was assayed by ORO staining at D4. Knockdown efficiency was assessed by RT-qPCR. Results are expressed relative to the indicated control in non-treated preadipocytes arbitrarily set to 1.

Supplemental Figure 8. 3T3-L1 cells adipogenesis is also altered by transfection of a LNA gapmer targeting *Paral1* similarly to unrelated siRNA. A) Validation of *Paral1* knockdown. 3T3-L1 cells were transfected with control (Ctrl-LNA) or *Paral1*-targeting LNA (*Paral1*-LNA) at D0. *Paral1* transcripts were assayed by RT-qPCR at D2 and results are expressed as the mean ± S.E.M. (n=3) relative to the *Paral1* RNA level in Ctrl-siRNA transfected preadipocytes arbitrarily set to 1. Values were compared using a t-test. * $p < 0.05$; ** $p < 0.01$; *** $p < 0.001$. B) Lipid accumulation in differentiated adipocytes. Oil Red O staining of 3T3L1 cells was performed at D8. C) Expression level variation of major contributors to the adipogenic program were extracted from microarray data and top ranking biological theme (using the Gene Ontology Biological Process functional annotation table, GO BP FAT) of down-regulated genes in *Paral1*-depleted cells.

Supplemental Figure 9. *Paral1* overexpression in 3T3-L1 cells does not promote adipogenesis. Clones of 3T3-L1 cells conditionally overexpressing either *PPARγ* or *Paral1* were generated as described in the Material and Methods section. After doxycycline stimulation and exposure (MDI+) or not (MDI-) to differentiation-inducing cocktail, the expression of *Paral1* (A), *PPARγ* (B) and of adiponectin (C, *AdipoQ*) was assayed by RT-qPCR. Results are expressed as the mean ± S.E.M. (n=3) relative to the gene expression level measured in control cells (pRetroX-empty) which was arbitrarily set to 1. Values were compared using a 1-way ANOVA. * $p < 0.05$; ** $p < 0.01$; *** $p < 0.001$. D) ORO staining of 3T3-L1 cells 7 days after initiation of adipogenic differentiation.

Supplemental Figure 10. *Paral1* deficiency alters the mature adipocyte phenotype. A) 3T3-L1 differentiation and transfection protocol. B) Validation of *Paral1* expression knockdown. 3T3-L1 cells were transfected with control (Ctrl-siRNA) or *Paral1*-targeting siRNA (*Paral1*-siRNA) at D7. *Paral1* transcripts were assayed by RT-qPCR at D9 and results are expressed as the mean ± S.E.M. (n=3) relative to the *Paral1* RNA level in Ctrl-siRNA transfected preadipocytes arbitrarily set to 1. Values were compared using a t-test. * $p < 0.05$; ** $p < 0.01$; *** $p < 0.001$. C) Knockdown of *Paral1* expression alters the expression of a specific subset of genes. The 3T3-L1 cell transcriptome at D9 was characterized by DNA microarray analysis as described in the Research Design and Methods section. GSEA was performed using the KEGG pathway gene sets. FC: fold change, red: up-regulated after *Paral1*-siRNA treatment, green: down-regulated after *Paral1/1*-siRNA treatment. D) Top ranking up- or down-regulated genes in Paral1-depleted mature 3T3-L1 cells-L1 adipocytes. E) Expression level variation of genes contributing to metabolic pathway regulation. Numbers indicate the fold change in Paral1-depleted adipocytes.

Supplemental Figure 11. Validation of the RBM14 antibody. Non-programed (none), mock-programed (pcDNA3) or programed (pcDNA3-*Rbm14*) reticulocyte lysates were analyzed by western blotting using the anti-RBM14 antibody. Apparent molecular masses are indicated on the left.

Supplemental Figure 12. Mapping the *Paral1*-RBM14 interaction. A) Structure of the vector used for in vitro transcription of *Paral1* and of its derivatives. B) RNA pulldown assays with *Paral1* fragments. Biotinylated *Paral1* fragments were coupled to magnetic streptavidin beads and incubated with 3T3-L1 cell extracts. Eluates were probed for RBM14 by western blotting.

Supplemental Figure 13. Secondary structure of *Paral1*. Predicted secondary structure of *Paral1* using the mFold software (1).

Supplemental Figure 14. *Rbm14* expression is efficiently downregulated by LNA Gapmers in *Paral1*-depleted cells at D2. *Rbm14* expression of Ctrl-LNA and *Rbm14*-LNA transfected 3T3-L1 cells at D0 was assessed by RT-qPCR at D2. Results are expressed as the mean ± S.E.M. (n=3) relative to the *Rbm14* RNA level in Ctrl-siRNA transfected preadipocytes arbitrarily set to 1. Values were compared using a t-test. * $p < 0.05$; ** $p < 0.01$; *** $p < 0.001$.

Supplemental Figure 15. Contribution of *Paral1*-associated paraspeckles components to adipocyte differentiation. Control (Ctrl-LNA) or LNA gapmers targeting *Pspc1*, *Sfpq* and *Nono* were transfected at D0 and lipid accumulation was assayed by ORO staining. Knockdown efficiency was assessed by RT-qPCR. Results are expressed as the mean ± S.E.M. (n=3) relative to the indicated control in non-treated preadipocytes arbitrarily set to 1. Values were compared using a t-test (*Paral1*) or 1-way ANOVA followed by a Dunnett post hoc test (*Pspc1*, *Sfpq* and *Nono*). * $p < 0.05$; ** $p < 0.01$; *** $p < 0.001$.

Supplemental Figure 16. Annotation and enrichment analysis of dysregulated genes in obese eWAT. A) Biological term enrichment of up- or down-regulated genes in eWAT from ob/ob mice. Gene lists were annotated with the GO BP FAT. Significantly enriched terms are shown as well as their p values. Red: up-regulated, green: down-regulated. B) Biological term enrichment of up- or down-regulated genes in eWAT from HFD fed mice. Gene lists were annotated with the GO BP FAT. Significantly enriched terms are shown as well as their p values. Red: up-regulated, green: down-regulated.

Supplemental Figure 17. *Pparγ and Rbm14* expression in obese mice eWAT. A) eWAT RNA from wild type (wt) or obese (ob/ob) C57Bl6/J mice were analysed for their content in *Pparγ and Rbm14* transcripts by RT-qPCR. Results are expressed as the mean ± S.E.M. (n=6-8) relative to the wild type level arbitrarily set to 1. Values were compared using a t-test. * $p < 0.05$; ** $p < 0.01$; *** $p < 0.001$. B) eWAT RNA from wild type (wt) C57Bl6/J fed either a chow diet (CD) or a high fat diet (HFD) were analysed for their content in *Pparγ and Rbm14* transcripts by RT-qPCR as above.

Supplemental Figure 18. TNF represses *Paral1* expression in differentiated 3T3-L1 cells. Differentiated 3T3-L1 cells were treated for 48 hours with the indicated dose of TNF. RNA was extracted and *Paral1* expression was assayed by RT-qPCR. Results are expressed relative to the *Paral1* RNA level in preadipocytes arbitrarily set to 1.

Supplemental Figure 19. Expression of *hPARAL1* (ENSG00000243961) in human tissues. Expression data were extracted from RNA-seq analysis of 16 human tissues (Illumina body map) available at the EMBL-EBI expression atlas web site (http://www.ebi.ac.uk/gxa/experiments/E-MTAB-513?geneQuery=%5B%7B%22value%22%3A%22ENSG00000243961%22%2C%22category%22%3A%22%22%7D%5D&serializedFilterFactors=).

Supplemental Figure 20. Downregulated pathways in obese patients. Gene set enrichment analysis (GSEA) score curves. Example of enrichment plots are shown for gene signatures identified by GSEA as being significantly downregulated in obese visWAT. Black bars represent the position of genes from the corresponding pathway in the ranked gene list. The enrichment score plot is shown in green.

BIBLIOGRAPHY

1. Zuker M: Mfold web server for nucleic acid folding and hybridization prediction. Nucleic Acids Res 2003;31:3406-3415

Firmin F. et al ; Supp.Fig.1

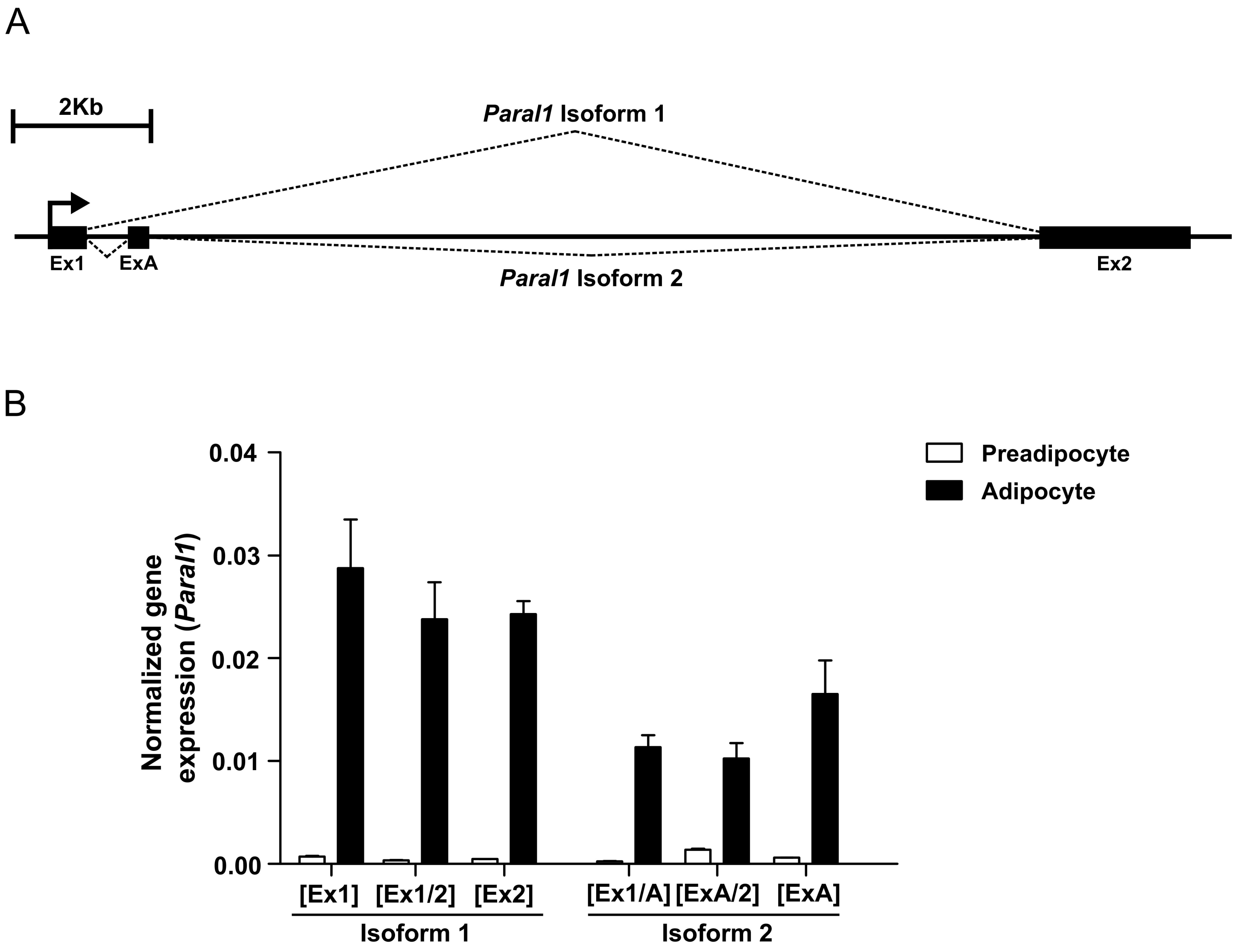

Firmin F. et al ; Supp.Fig.2

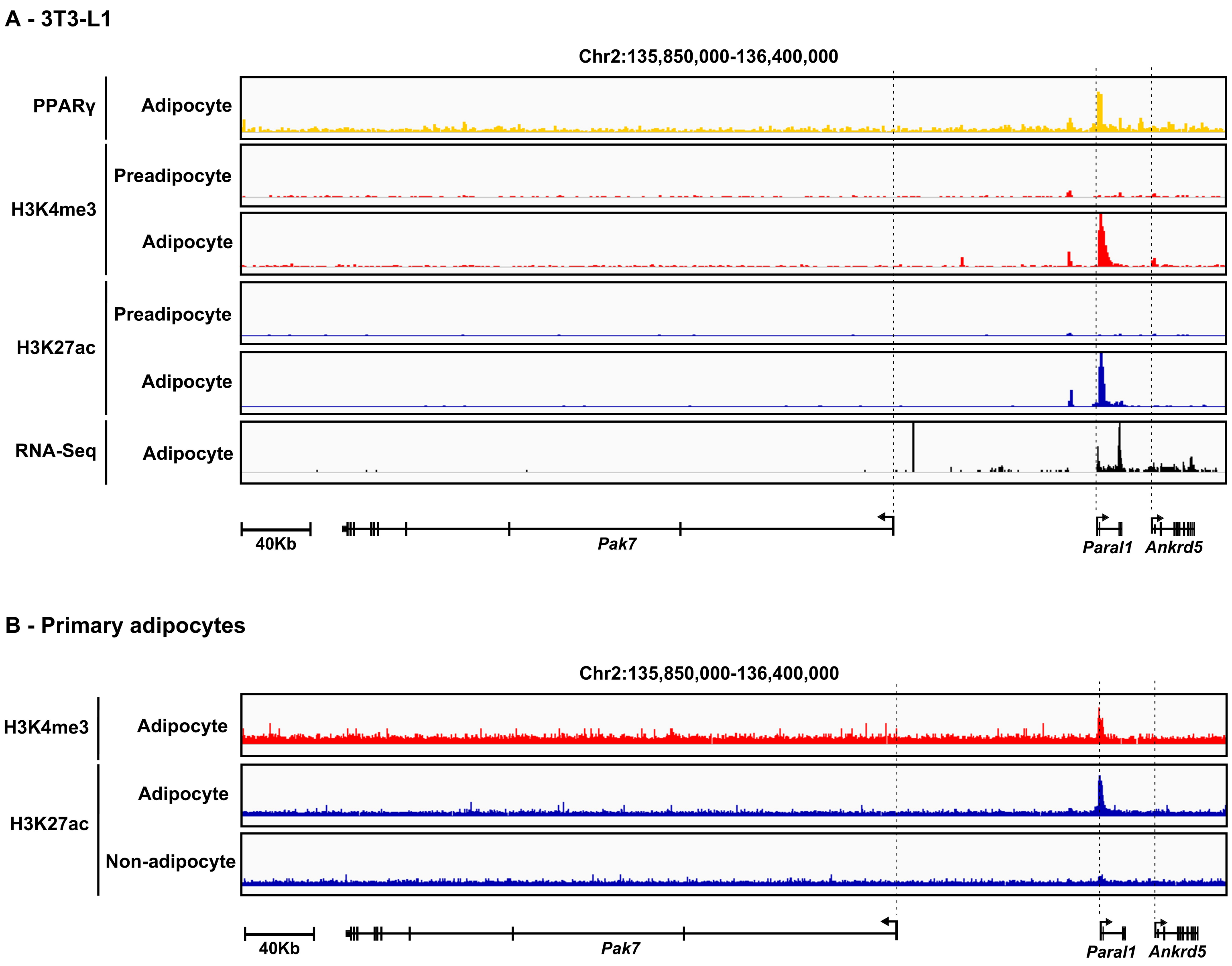

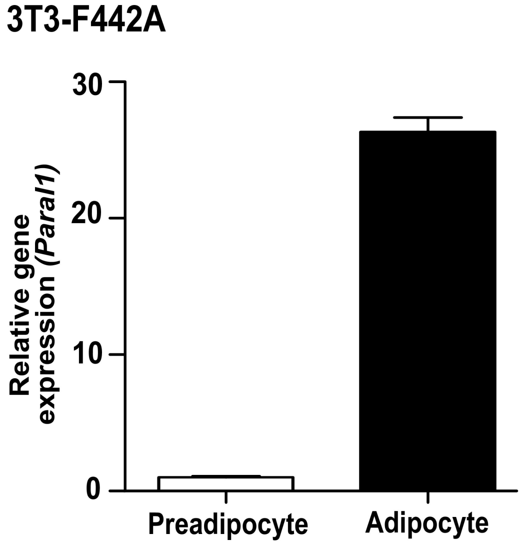
3T3-F442A
Relative gene expression (Paral1)
30
20
10
0
Preadipocyte
Adipocyte

Firmin F. et al ; Supp.Fig.4

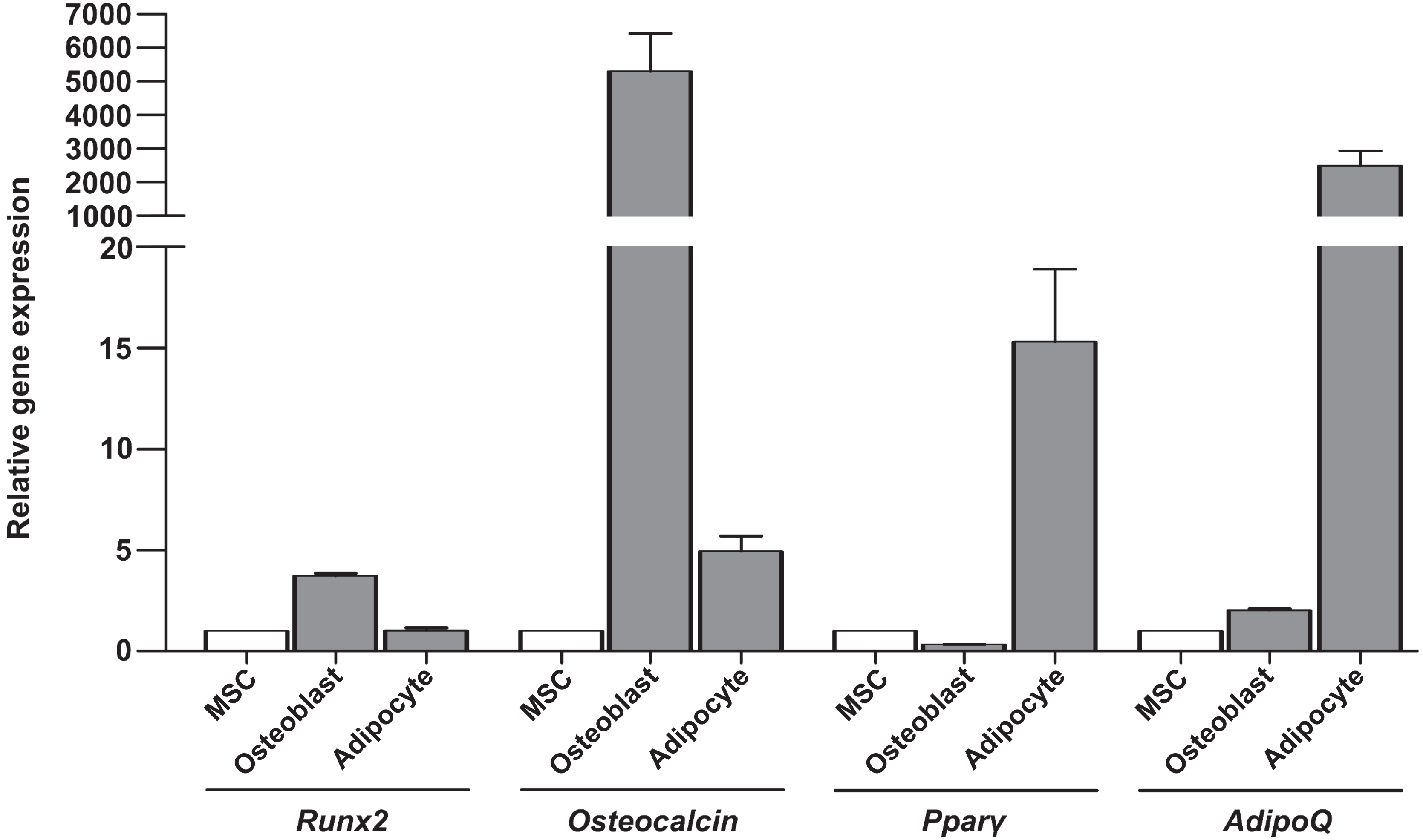

Firmin F. et al ; Supp.Fig.5

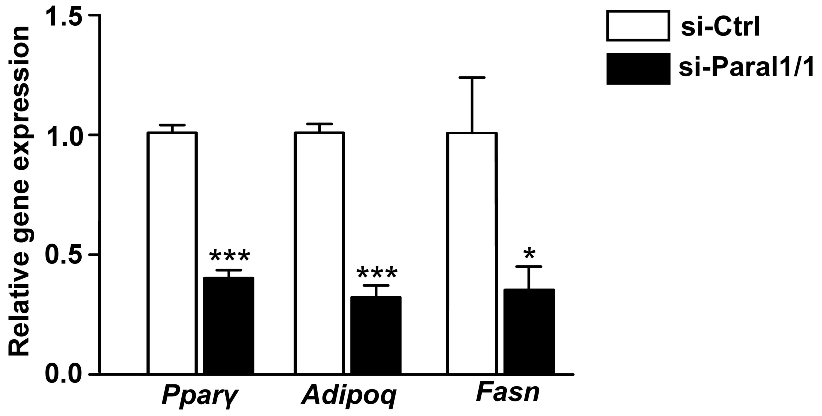

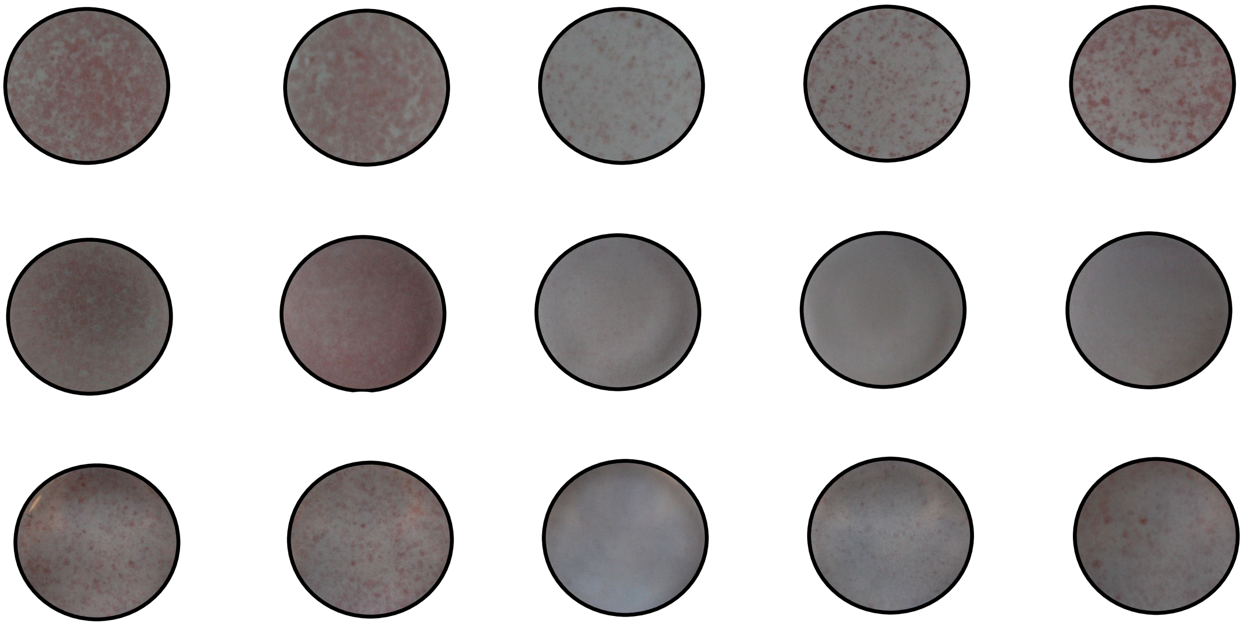

NT **Ctrl-siRNA** ***Pparγ*-siRNA** ***Paral1/1*-siRNA** ***Paral1/2*-siRNA**

Firmin F. et al ; Supp.Fig.7

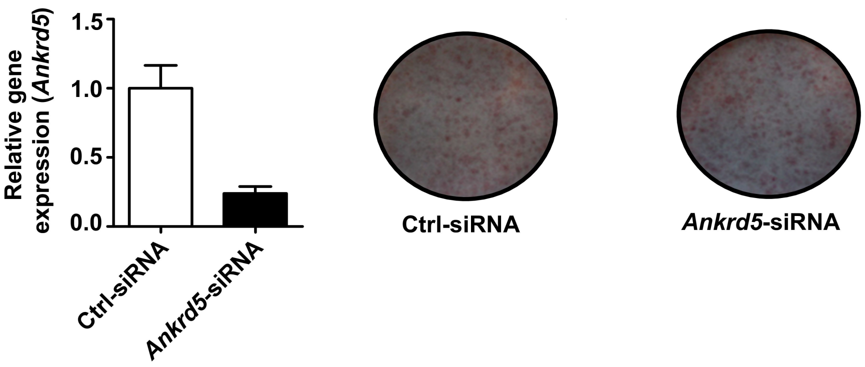

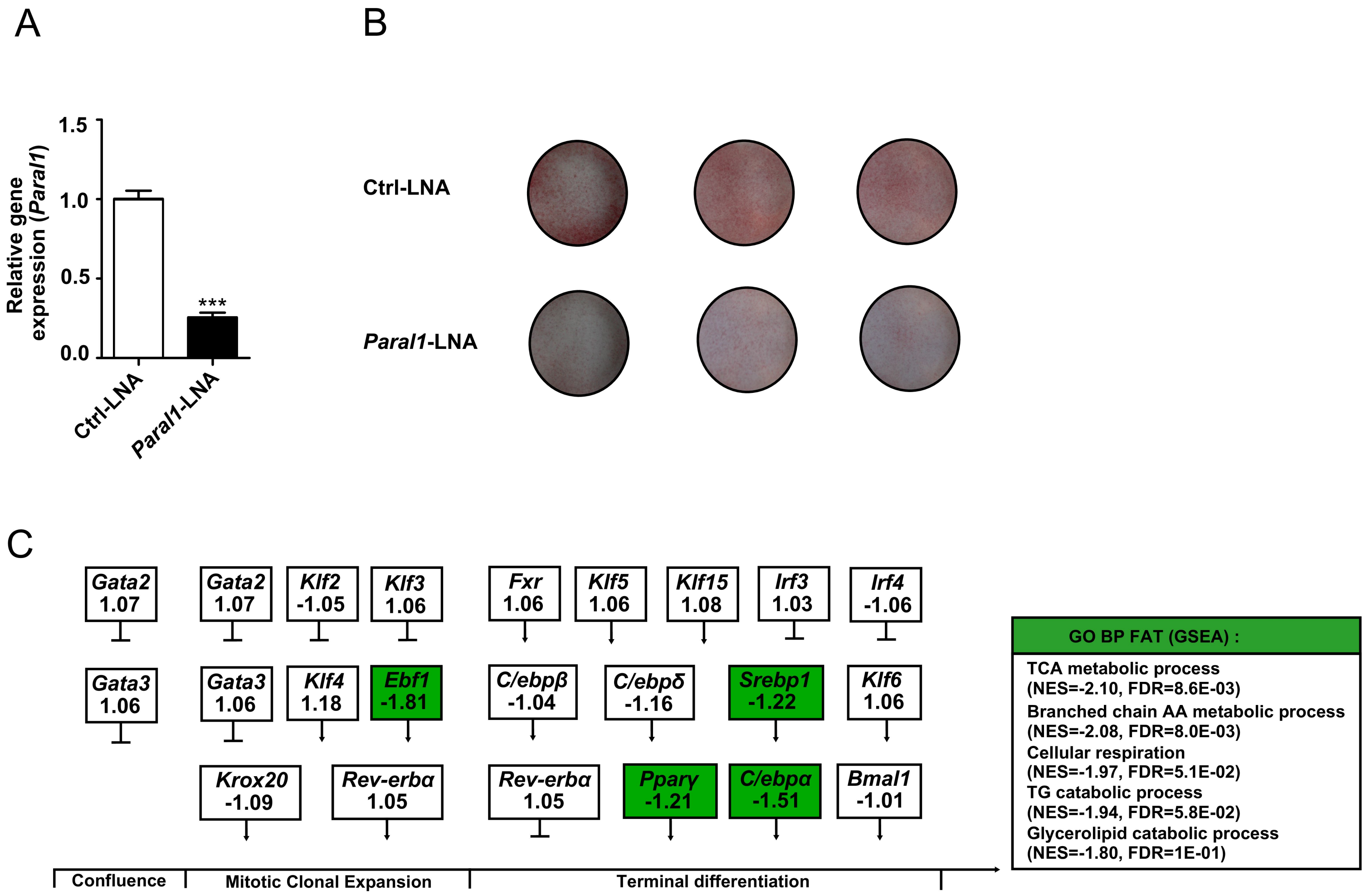
A
1.5
1.0
0.5
0.0
Relative gene expression (Paral1)
***
Ctrl-LNA
Paral1-LNA
B
Ctrl-LNA
Paral1-LNA
C
Gata2 1.07
Gata3 1.06
Gata2 1.07
Klf2 -1.05
Klf3 1.06
Gata3 1.06
Klf4 1.18
Ebf1 -1.81
Krox20 -1.09
Rev-erbα 1.05
Fxr 1.06
Klf5 1.06
Klf15 1.08
Irf3 1.03
Irf4 -1.06
C/ebpβ -1.04
C/ebpδ -1.16
Srebp1 -1.22
Klf6 1.06
Rev-erbα 1.05
Pparγ -1.21
C/ebpα -1.51
Bmal1 -1.01
Confluence
Mitotic Clonal Expansion
Terminal differentiation
GO BP FAT (GSEA) :
TCA metabolic process
(NES=-2.10, FDR=8.6E-03)
Branched chain AA metabolic process
(NES=-2.08, FDR=8.0E-03)
Cellular respiration
(NES=-1.97, FDR=5.1E-02)
TG catabolic process
(NES=-1.94, FDR=5.8E-02)
Glycerolipid catabolic process
(NES=-1.80, FDR=1E-01)

Firmin F. et al ; Supp.Fig.9

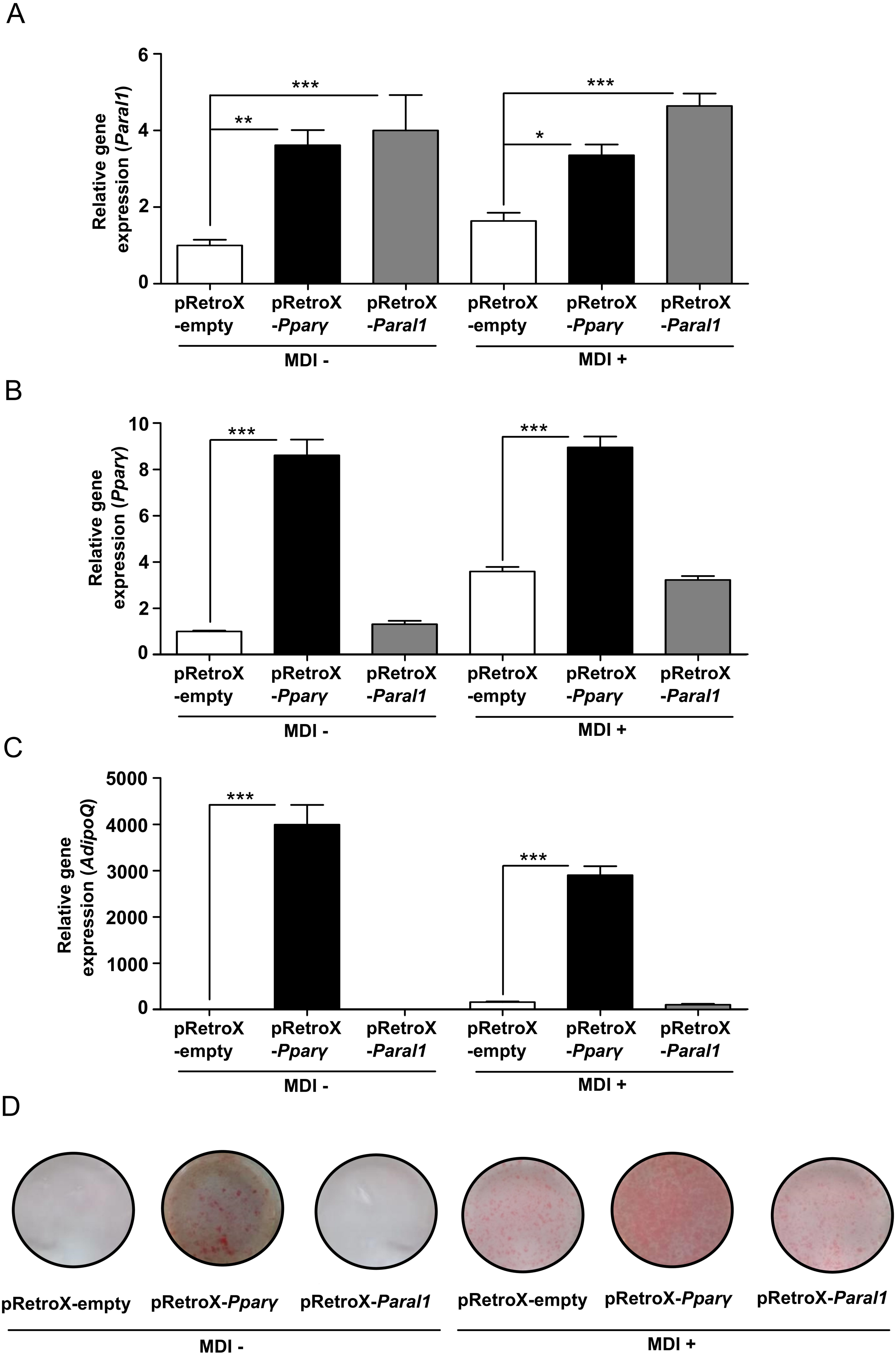

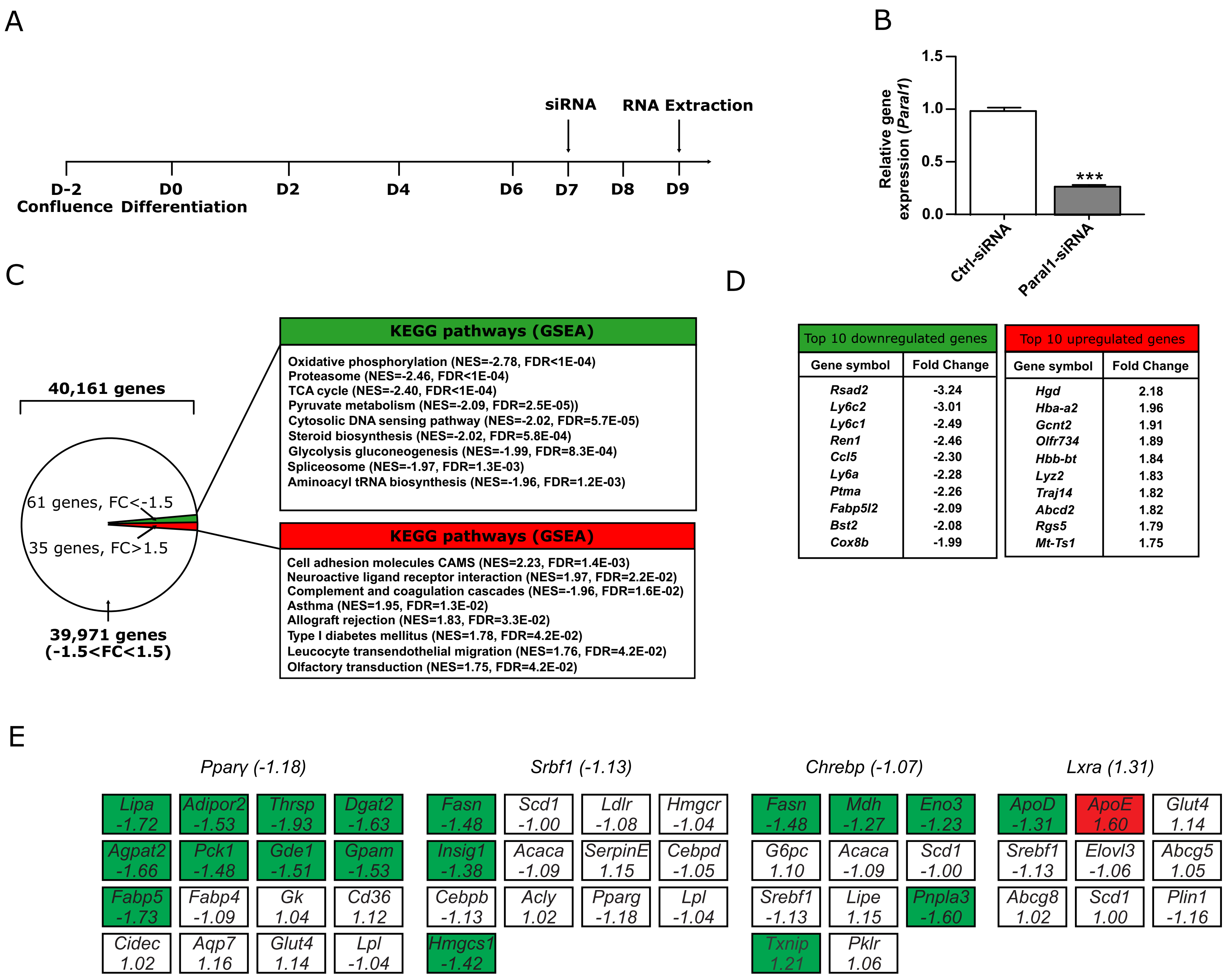
A
siRNA
RNA Extraction
D-2
D0
D2
D4
D6
D7
D8
D9
Confluence
Differentiation
B
Relative gene expression (Paral1)
1.5
1.0
0.5
0.0
***
Ctrl-siRNA
Paral1-siRNA
C
40,161 genes
61 genes, FC<-1.5
35 genes, FC>1.5
39,971 genes (-1.5<FC<1.5)
KEGG pathways (GSEA)
Oxidative phosphorylation (NES=-2.78, FDR<1E-04)
Proteasome (NES=-2.46, FDR<1E-04)
TCA cycle (NES=-2.40, FDR<1E-04)
Pyruvate metabolism (NES=-2.09, FDR=2.5E-05))
Cytosolic DNA sensing pathway (NES=-2.02, FDR=5.7E-05)
Steroid biosynthesis (NES=-2.02, FDR=5.8E-04)
Glycolysis gluconeogenesis (NES=-1.99, FDR=8.3E-04)
Spliceosome (NES=-1.97, FDR=1.3E-03)
Aminoacyl tRNA biosynthesis (NES=-1.96, FDR=1.2E-03)
KEGG pathways (GSEA)
Cell adhesion molecules CAMS (NES=2.23, FDR=1.4E-03)
Neuroactive ligand receptor interaction (NES=1.97, FDR=2.2E-02)
Complement and coagulation cascades (NES=-1.96, FDR=1.6E-02)
Asthma (NES=1.95, FDR=1.3E-02)
Allograft rejection (NES=1.83, FDR=3.3E-02)
Type I diabetes mellitus (NES=1.78, FDR=4.2E-02)
Leucocyte transendothelial migration (NES=1.76, FDR=4.2E-02)
Olfactory transduction (NES=1.75, FDR=4.2E-02)
D
Top 10 downregulated genes
Gene symbol | Fold Change
Rsad2 -3.24
Ly6c2 -3.01
Ly6c1 -2.49
Ren1 -2.46
Ccl5 -2.30
Ly6a -2.28
Ptma -2.26
Fabp5l2 -2.09
Bst2 -2.08
Cox8b -1.99
Top 10 upregulated genes
Gene symbol | Fold Change
Hgd 2.18
Hba-a2 1.96
Gcnt2 1.91
Olfr734 1.89
Hbb-bt 1.84
Lyz2 1.83
Traj14 1.82
Abcd2 1.82
Rgs5 1.79
Mt-Ts1 1.75
E
Pparγ (-1.18)
Lipa -1.72
Adipor2 -1.53
Thrsp -1.93
Dgat2 -1.63
Agpat2 -1.66
Pck1 -1.48
Gde1 -1.51
Gpam -1.53
Fabp5 -1.73
Fabp4 -1.09
Gk 1.04
Cd36 1.12
Cidec 1.02
Aqp7 1.16
Glut4 1.14
Lpl -1.04
Srbf1 (-1.13)
Fasn -1.48
Scd1 -1.00
Ldlr -1.08
Hmgcr -1.04
Insig1 -1.38
Acaca -1.09
SerpinE 1.15
Cebpd -1.05
Cebpb -1.13
Acly 1.02
Pparg -1.18
Lpl -1.04
Hmgcs1 -1.42
Chrebp (-1.07)
Fasn -1.48
Mdh -1.27
Eno3 -1.23
G6pc 1.10
Acaca -1.09
Scd1 -1.00
Srebf1 -1.13
Lipe 1.15
Pnpla3 -1.60
Txnip 1.21
Pklr 1.06
Lxra (1.31)
ApoD -1.31
ApoE 1.60
Glut4 1.14
Srebf1 -1.13
Elovl3 -1.06
Abcg5 1.05
Abcg8 1.02
Scd1 1.00
Plin1 -1.16

A

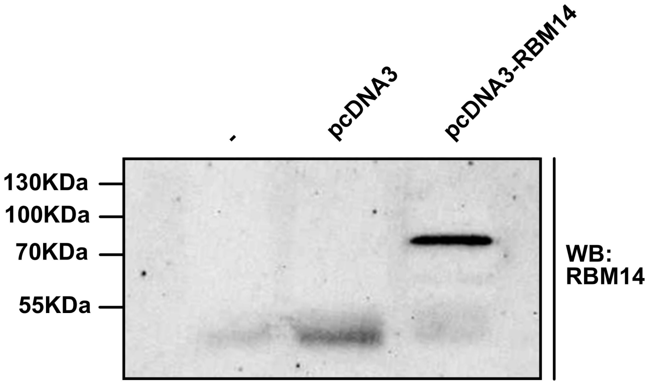

Firmin F. et al ; Supp.Fig.12

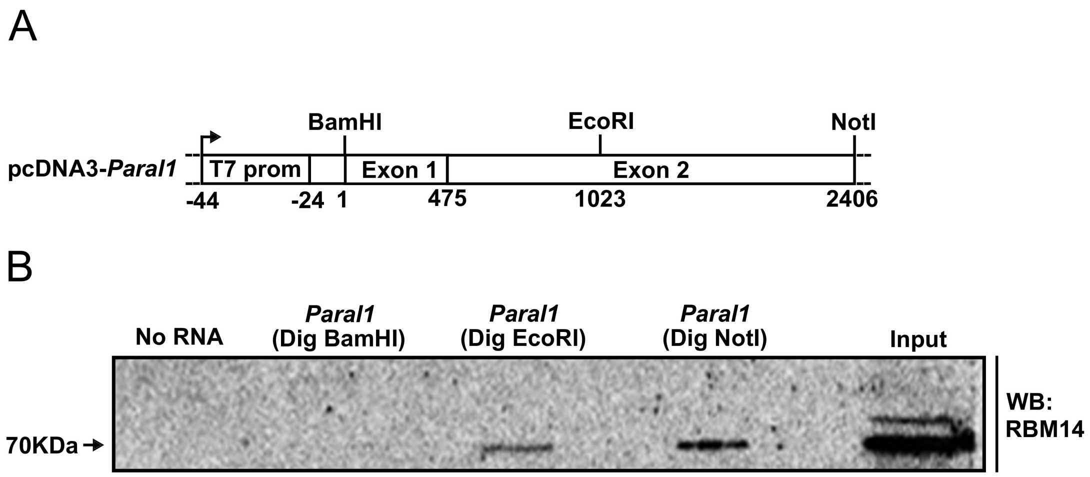

Firmin F. et al ; Supp.Fig.13

Paral1 (mFold)

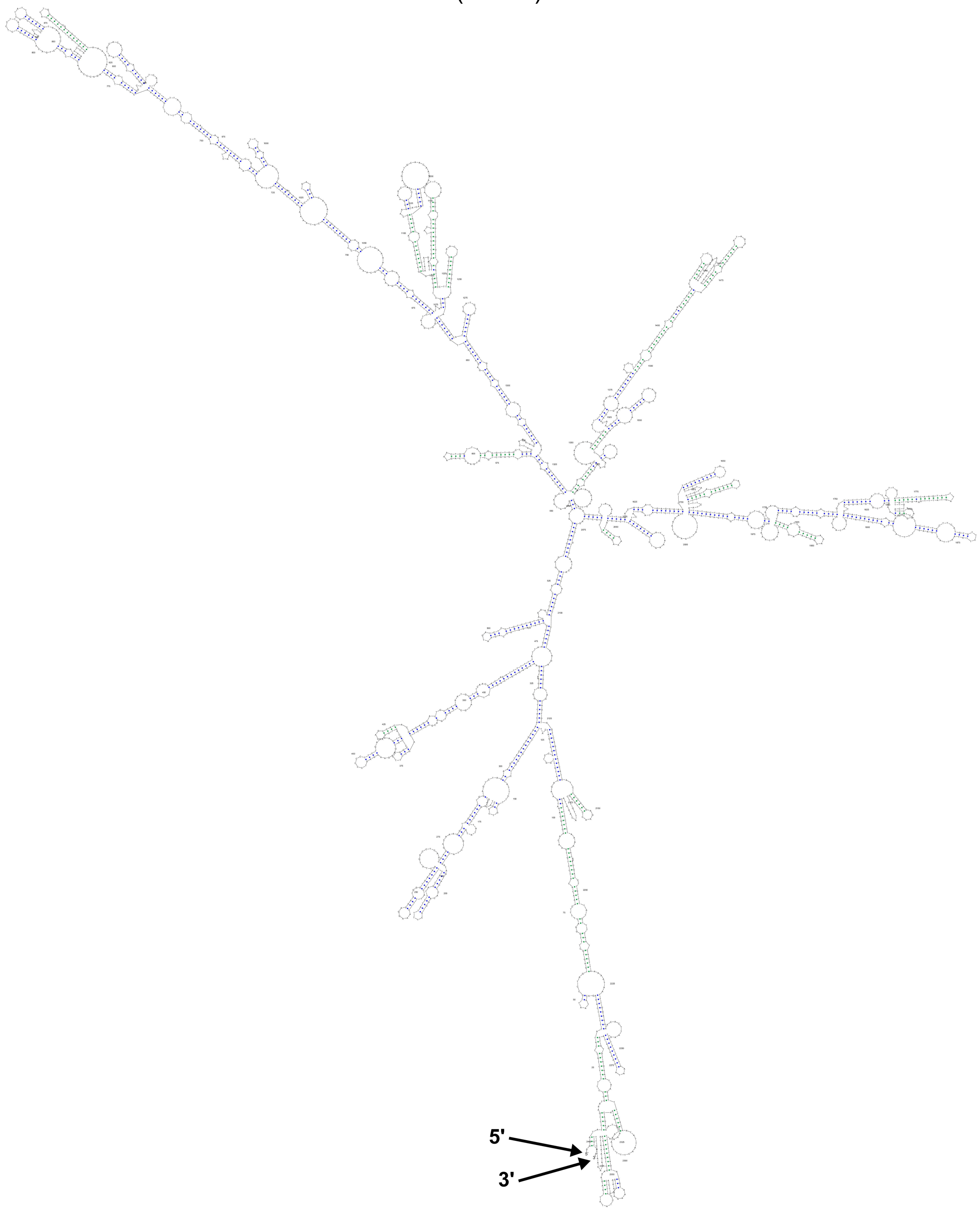

ΔG°_{37} = -614.30

Firmin F. et al ; Supp.Fig.14

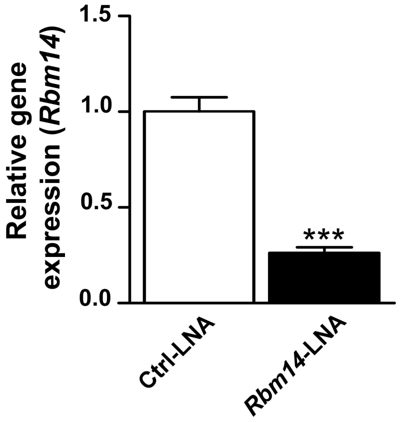

Firmin F. et al ; Supp.Fig.15

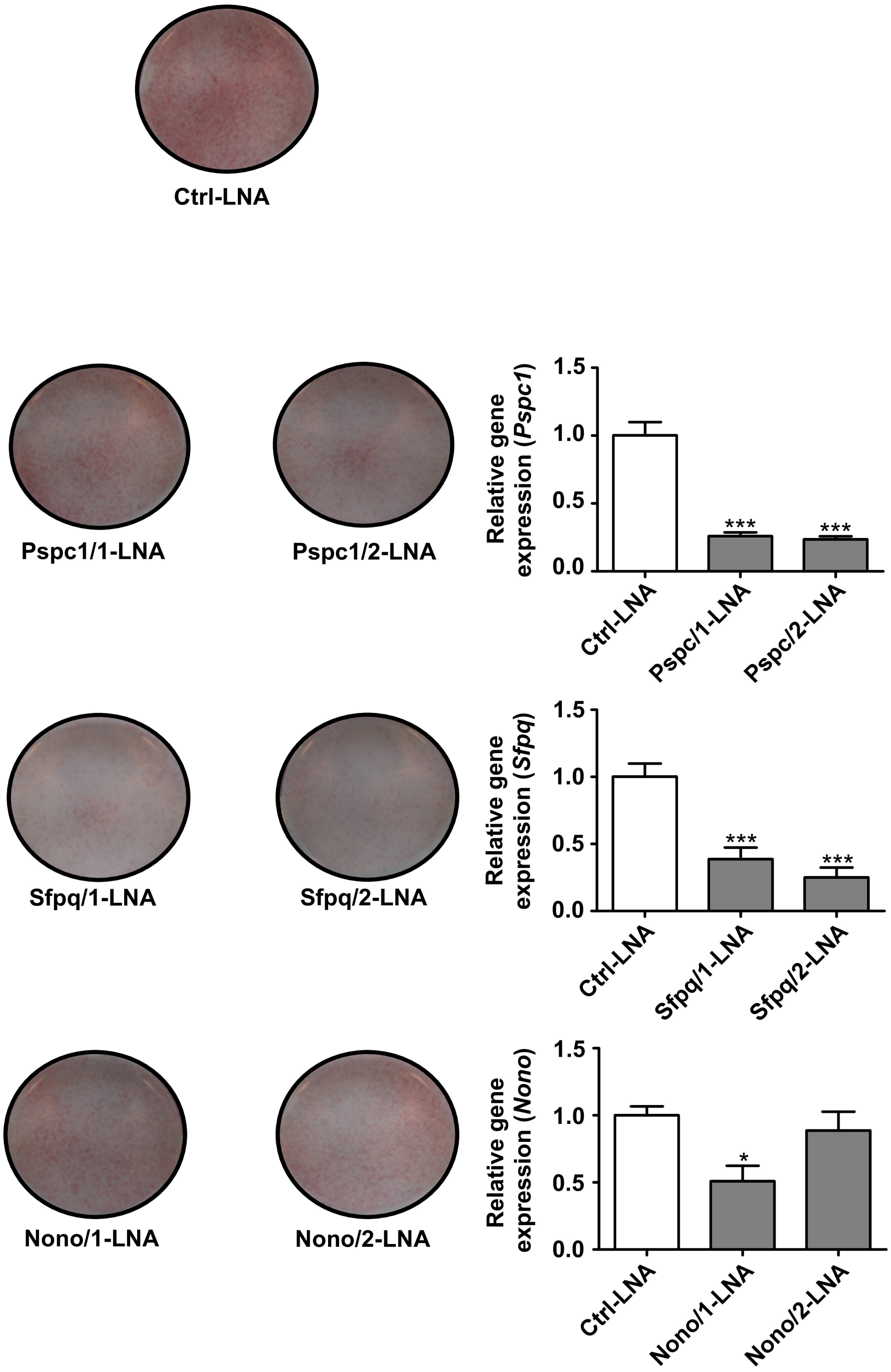

Firmin F. et al ; Supp.Fig.16

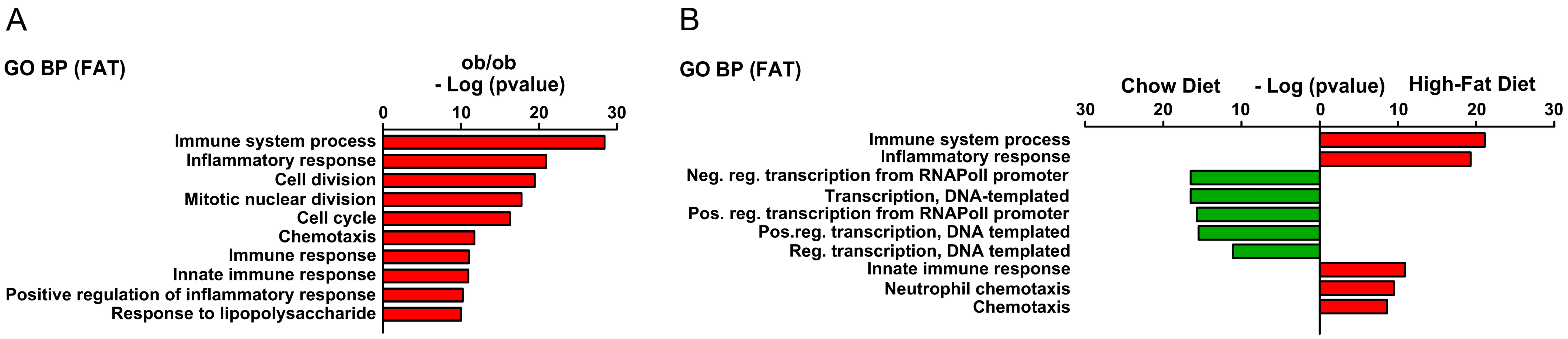

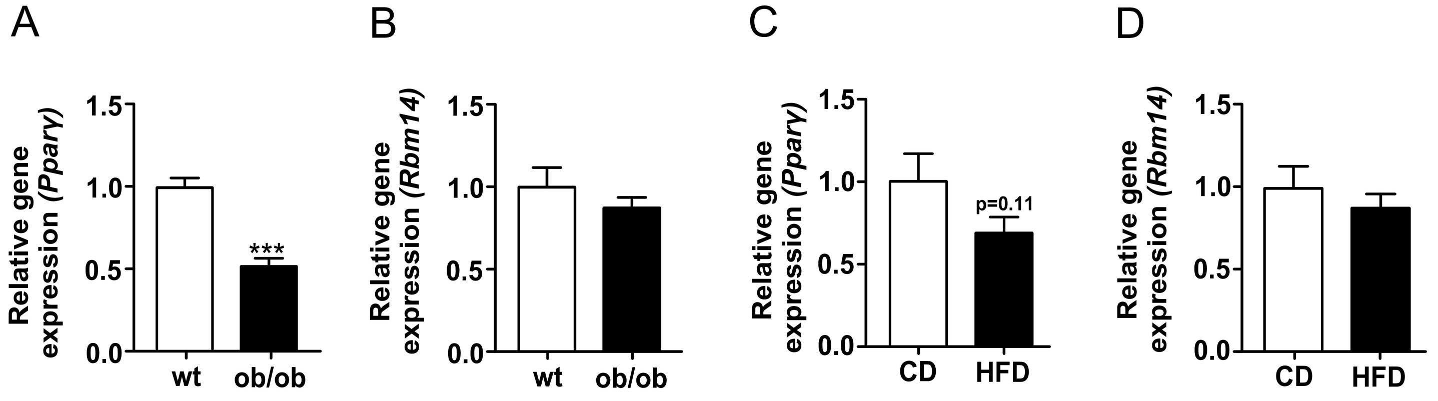
A
B
C
D
Relative gene expression (Pparγ)
Relative gene expression (Rbm14)
1.5
1.0
0.5
0.0
***
p=0.11
wt
ob/ob
CD
HFD

Firmin F. et al ; Supp.Fig. 18

3T3-L1

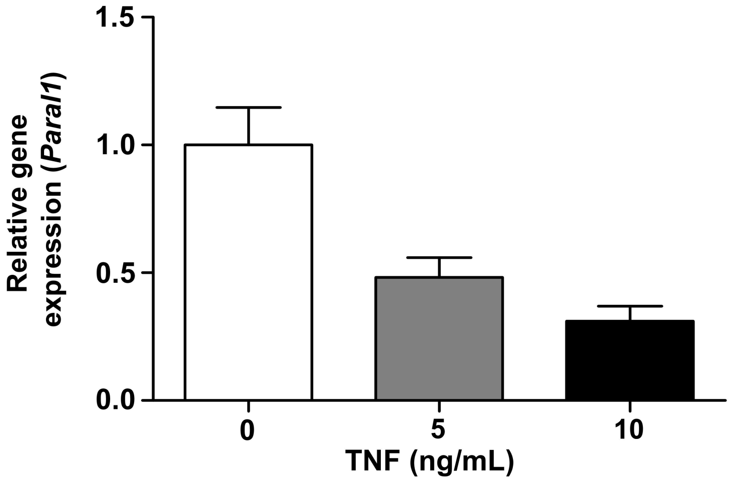

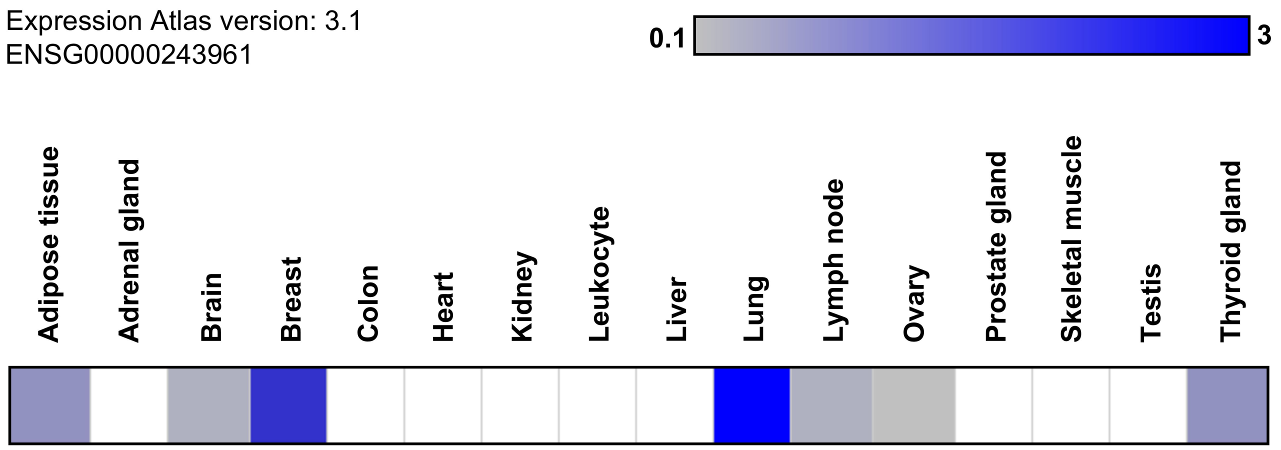
Expression Atlas version: 3.1
ENSG00000243961
0.1
3
Adipose tissue
Adrenal gland
Brain
Breast
Colon
Heart
Kidney
Leukocyte
Liver
Lung
Lymph node
Ovary
Prostate gland
Skeletal muscle
Testis
Thyroid gland

Firmin F. et al ; Supp.Fig.20

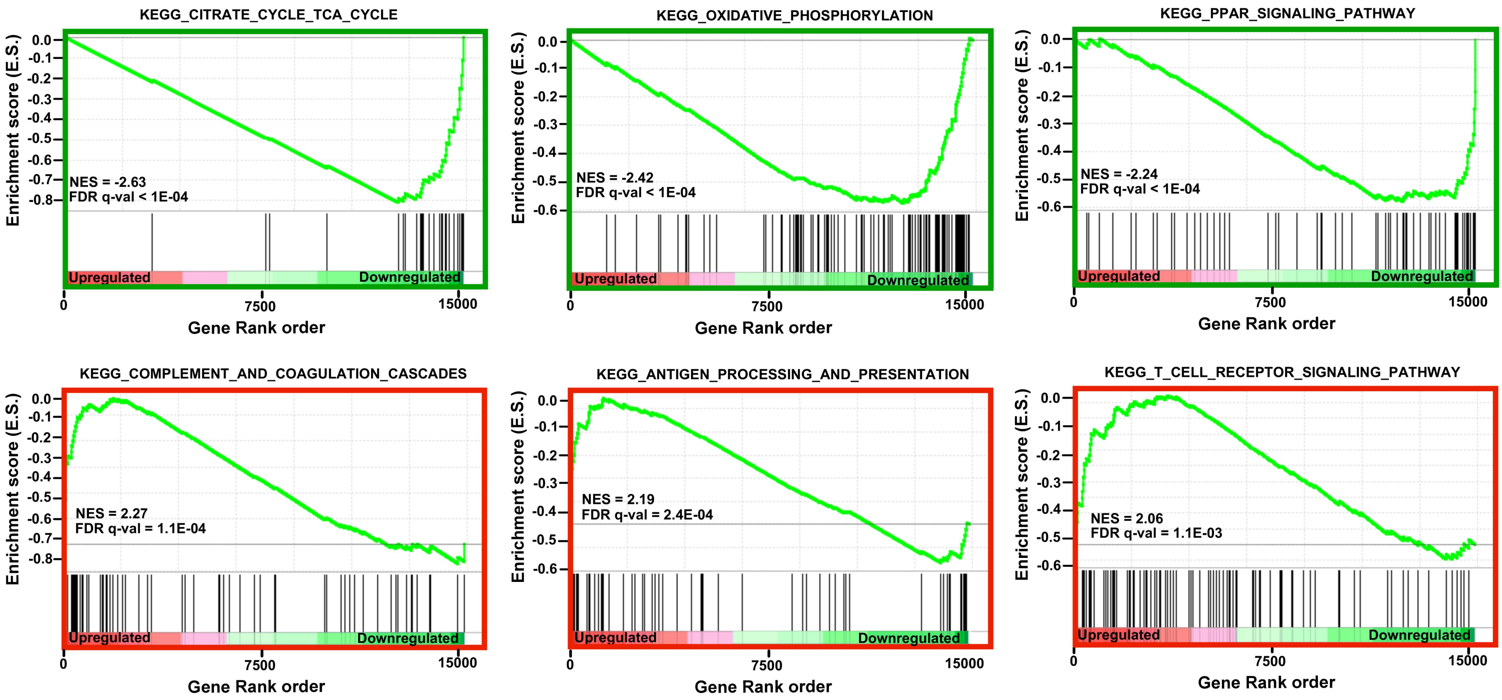

Uncropped version of Western blot (Figure 5C)

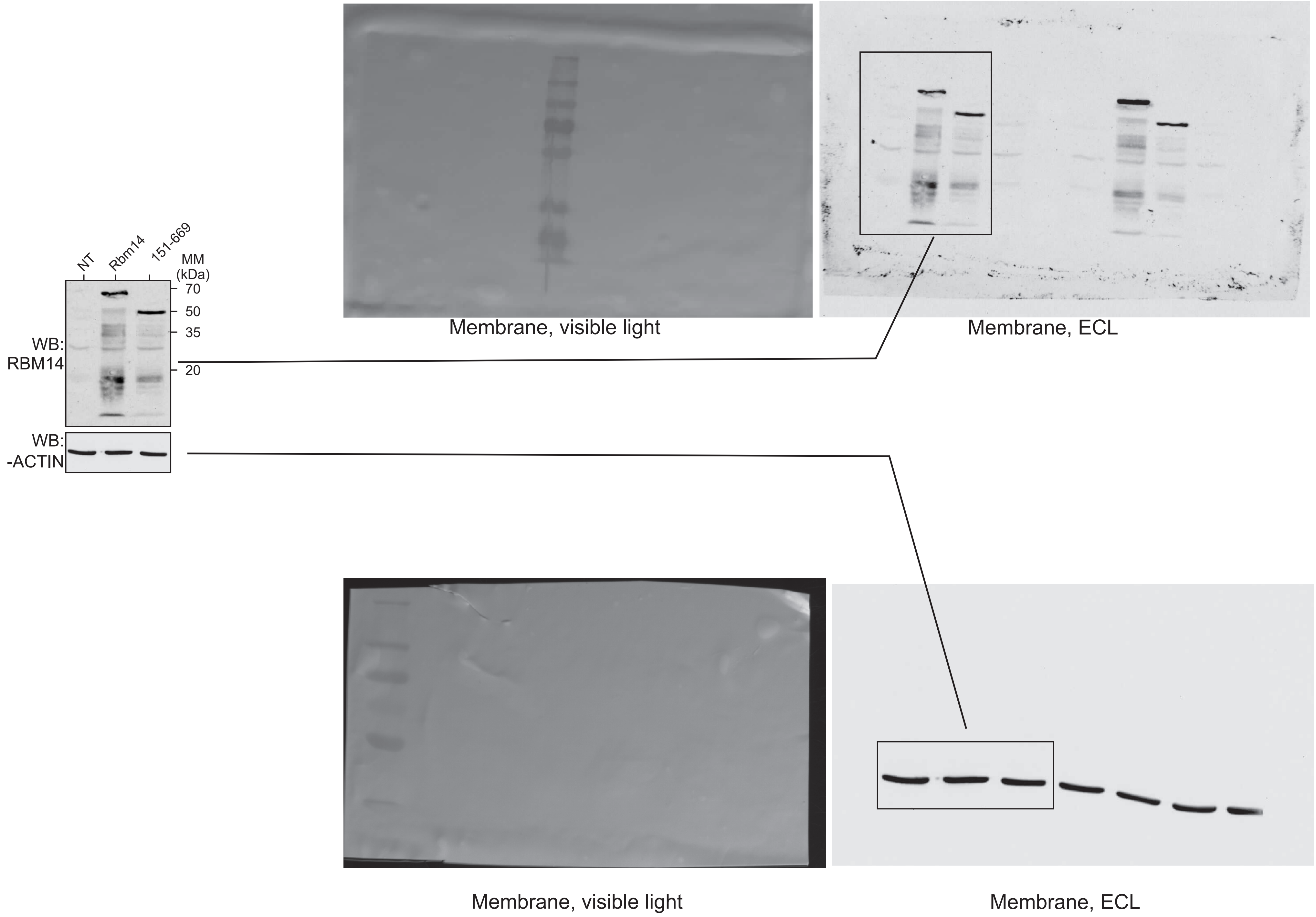

Supplement: Supplementary file 1 — Supplemental information [file 41598_2017_14570_MOESM1_ESM.pdf]
